# 4-Octyl itaconate alleviates endothelial cell inflammation and barrier dysfunction in LPS-induced sepsis via modulating TLR4/MAPK/NF-κB signaling: 4-Octyl itaconate alleviates endothelial dysfunction

**DOI:** 10.1186/s10020-025-01160-2

**Published:** 2025-06-16

**Authors:** Rong Li, Yu Ma, Haoran Wu, Xiao Zhang, Nianhui Ding, Zhichao Li, Xin Hu, Jiajia Rao, Yiting Zhou, Liqun Wang, Ying Wan, Yan Yang, Jianbo Wu, Xiaoqin Zhang, Chunxiang Zhang

**Affiliations:** 1 Basic Medicine Research Innovation Center for Cardiometabolic Diseases, Ministry of Education; Luzhou Municipal Key Laboratory of Thrombosis and Vascular Biology; Laboratory for Cardiovascular Pharmacology, Department of Pharmacology, School of Pharmacy, Southwest Medical University, Luzhou, 646000 Sichuan China; 2 Department of Cardiology, the Affiliated Hospital of Southwest Medical University, Luzhou 646000, Sichuan, China; Department of Cardiology, Guangyuan Hospital of Traditional Chinese Medicine, Guangyuan, 628000 Sichuan China; 3 School of Basic Medical Science, Southwest Medical University, Luzhou, Sichuan 646000 China; 4 Anesthesiology and Critical Care Medicine Key Laboratory of Luzhou, The Affiliated Hospital of Southwest Medical University, Luzhou, Sichuan 646000 China; 5 Institute of Cardiovascular Research, Southwest Medical University, Luzhou, 646000 Sichuan China; 6 Department of Cardiology, the Affiliated Hospital of Southwest Medical University; Nucleic Acid Medicine of Luzhou Key Laboratory; Key Laboratory of Medical Electrophysiology; Ministry of Education & Medical Electrophysiological Key Laboratory of Sichuan Province; Institute of Cardiovascular Research, Southwest Medical University, Luzhou, 646000 Sichuan China

**Keywords:** Sepsis, Acute lung injury, Endothelial cell, 4-Octyl Itaconate, Vascular injury

## Abstract

**Aim:**

Sepsis-induced vascular injury is a major contributor to the high mortality rate of sepsis. However, effective treatments remain elusive due to limited knowledge regarding the underlying molecular mechanisms. Itaconic acid, an endogenous metabolite, involved in multiple inflammatory diseases, but its role in sepsis-induced vascular injury remains unclear. The current study investigates the effect of 4-octyl itaconate (4-OI), a cell-permeable derivative of itaconic acid, on sepsis-induced vascular injury and organ damage.

**Methods and results:**

An in vitro cell model was established by treating human umbilical vein endothelial cells (HUVECs) with lipopolysaccharide (LPS). Quantitative reverse transcription polymerase chain reaction (qRT-PCR) and enzyme-linked immunosorbent assay (ELISA) revealed that 4-OI inhibited the LPS-induced increases in TNF-α, IL-6, and IL-1β levels. Cellular reactive oxygen species (ROS) levels, measured using the fluorescent probe DCFH-DA, mitochondrial ROS (mtROS) levels, measured by MitoSOX, and mitochondrial membrane potential (ΔΨ), detected by the fluorescent indicator JC-1, were all reduced following 4-OI treatment. Additionally, mtDNA release, detected by qRT-PCR, were decreased. Mitochondrial morphology, assessed by PK Mito Orange, was preserved by 4-OI treatment. Furthermore, 4-OI suppressed HUVECs apoptosis and pyroptosis, as detected by TUNEL staining and western blotting. 4-OI treatment also significantly inhibited LPS-induced cell adhesion, as shown in THP-1 attachment assay, by decreasing ICAM-1 and VCAM-1 expression. Cell permeability, determined by FITC-Dx-70 leakage, revealed that 4-OI effectively suppressed LPS-induced increases in cell permeability. Furthermore, 4-OI inhibited LPS-induced phosphorylation and internalization of VE-cadherin protein, preserving the adhesion junctions between endothelial cells. Network pharmacology and molecular docking analysis suggested the involvement of TLR4/MAPK/NF-κB signaling pathway as a key mechanism by which 4-OI ameliorated sepsis-induced vascular cell inflammation and injury, which was confirmed by western blotting. The in vitro results were subsequently verified in vivo in an LPS-induced sepsis mouse model. 4-OI pretreatment substantially decreased inflammatory cytokine levels in serum and lung tissues, inhibited pulmonary oedema and pulmonary vascular leakage, as evidenced by the wet-to-dry weight ratio and Evans blue staining of lung tissues, and alleviated tissue damage, as shown by histological analysis. Survival analysis indicated that 4-OI post-sepsis treatment improved the overall survival rate in LPS-induced ALI mice.

**Conclusion:**

4-OI protects against sepsis-induced vascular injury and tissue damage by suppressing endothelial inflammation, oxidative stress, and preserving endothelial barrier integrity.

**Graphical abstract:**

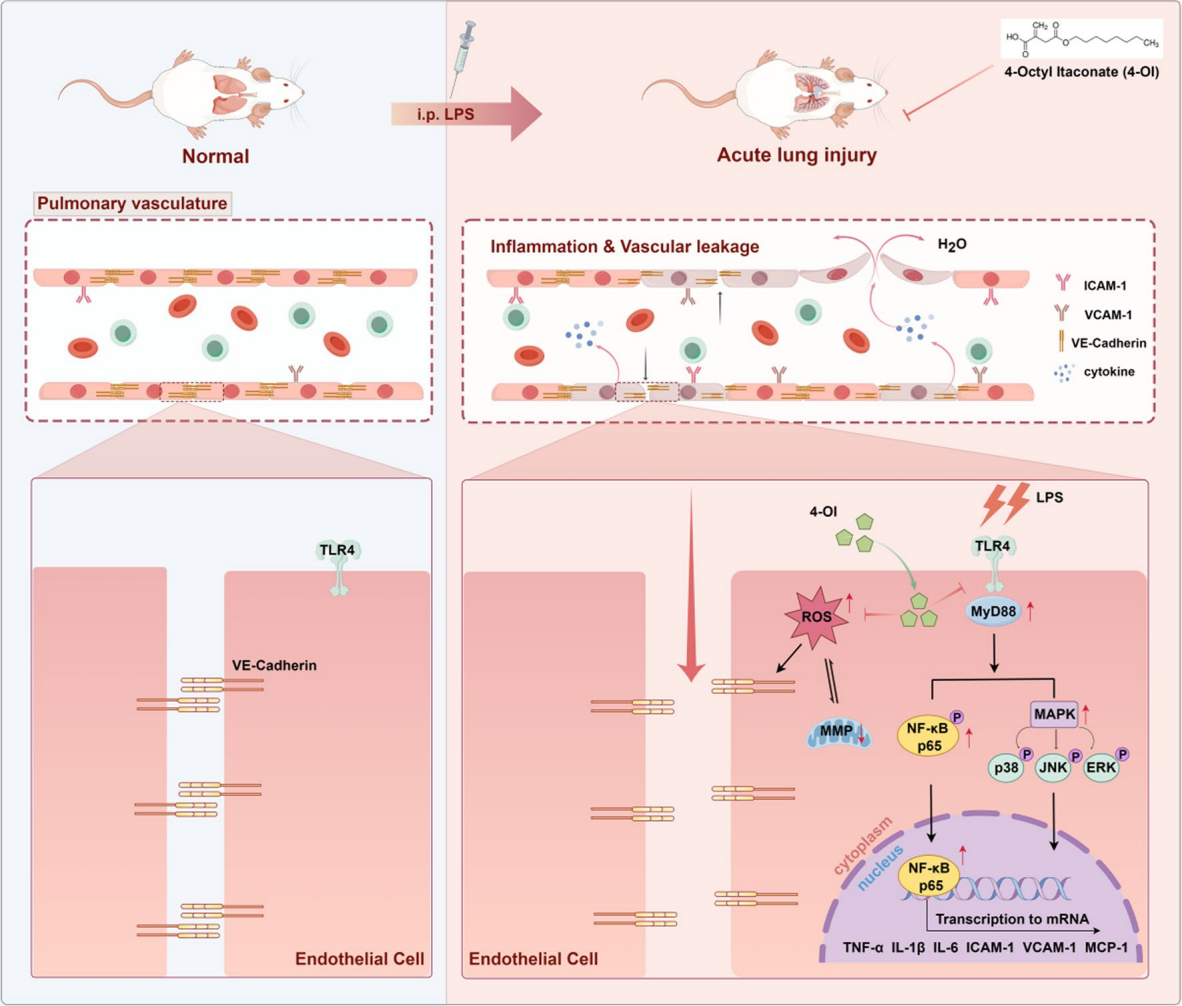

**Supplementary Information:**

The online version contains supplementary material available at 10.1186/s10020-025-01160-2.

## Translational perspective

Sepsis-induced vascular injury significantly contributes to sepsis-related organ damage, such as acute lung injury (ALI), and to the high mortality rate of sepsis. Our study demonstrated the effectiveness of 4-OI (4-Octyl Itaconate) in reducing the inflammatory response and oxidative stress caused by lipopolysaccharide (LPS), enhancing the connections between endothelial cells, and maintaining the integrity of the endothelial barrier in both in vivo and in vitro models. The inhibition of the TLR4/MAPK/NF-κB signaling pathway could play a crucial role in this protective mechanism. Thus, 4-OI appears to be a promising therapeutic agent for sepsis.

**Supplementary Information:**

The online version contains supplementary material available at 10.1186/s10020-025-01160-2.

## Introduction

Sepsis is a fatal condition triggered by microbial infection and characterized by an amplified host inflammatory responses and organ damage, including acute lung injury (ALI), which is characterized by dysregulated inflammatory responses, increased pulmonary vascular permeability, and diffuse alveolar damage (Yang et al. 2020, 2022). Interactions between activated alveolar macrophages (AMs) and pulmonary endothelial cells (PECs), underlie the resulting vascular injury and are crucial for the occurrence of ALI (Osorio-Valencia and Zhou 2024).Consistant with this, severe infection and septicemia appear to lead to pulmonary microangiopathy, accompanied by the recruitment of leucocytes and the overproduction of inflammatory cytokines and reactive oxygen species (ROS), which in turn lead to further damage to PECs and a weakening of the endothelial barrier (Lou et al. 2021; Prado et al. 2023).

In addition, inflammatory cytokines and ROS can trigger oxidative stress in endothelial cells by damaging lipids, proteins, and DNA molecules, ultimately resulting in apoptosis and increased vascular permeability (Park et al. 2018). Therefore, controlling the inflammatory response, ROS accumulation, and PEC injury appears crucial for preventing ALI during sepsis (Chen et al. 2020).

Recent studies indicates that metabolites of the tricarboxylic acid cycle (TCA), specifically succinic acid, citric acid, and fumaric acid, perform important functions as signaling molecules that regulate the immune response (Choi et al. 2021). Manipulation of these endogenous compounds may offer a potential strategy for disease management and prevention. Moreover, Itaconic acid, an endogenous metabolite in the TCA, is notably increased when macrophages are activated (Strelko et al. 2011). Supporting a role for these metabolites, recent studies have highlighted the influence of itaconic acid and its derivatives in modulating inflammation, antimicrobial properties, and immunomodulation in diverse animal models (Elmore et al. 2021; Kuo et al. 2020; Bambouskova et al. 2018; Mills et al. 2018). Thus, 4-Octyl itaconate (4-OI), a cell permeable synthetic derivative of itaconate, has been shown to potentially protect mice from acute liver failure by attenuating inflammation, oxidative stress, and apoptosis (Li et al. 2021). Additionally, 4-OI has been shown to be effective in ameliorating skin lesions in mice with psoriasis by targeting the IL-17–IκBζ axis, extending the survival of mice with renal ischemia/reperfusion injury (IRI) mice by promoting Nrf2 nuclear translocation (Zhu et al. 2021), and limiting microglial activation and neuroinflammation in mice with experimental autoimmune encephalomyelitis (EAE) (Kuo et al. 2020). 

4-OI has further been demonstrated to decrease STING-dependent type I interferon synthesis by activating Nrf2 via an unidentified mechanism (Olagnier et al. 2018). Liu et al. emphasized itaconate is effective in treating COVID-19 (Sanchez-Cerrillo et al. 2020), and Olagnier et al. reported that 4-OI can directly block SARS-CoV-2 replication (Olagnier et al. 2020). Nonetheless, the question of whether itaconic acid and its derivatives can ameliorate pulmonary endothelial barrier dysfunction in lipopolysaccharide (LPS)-induced ALI remains unanswered.

The present study therefore aimed to determine the effect of 4-OI on the endothelial dysfunction in sepsis and to explore candidate underlying mechanisms. Collectively, the results suggested that 4-OI inhibits the expression of Toll-like receptor 4 (TLR4) in endothelial cells and associated downstream signaling pathways, reduces inflammation, oxidative stress and vascular permeability, and ultimately protects against pulmonary endothelial barrier and lung tissue damage in LPS-induced ALI.

## Methods

### Cell culture and treatment

Human umbilical vein endothelial cells (HUVECs) were obtained from ScienCell Research Laboratories (San Diego, CA, USA). The cells were grown in endothelial cell medium (ECM) supplemented with 5% fetal bovine serum (FBS) and 1% endothelial growth factor, and maintained in a humidified environment with 5% CO_2_ at 37℃. Cells in the exponential growth phase were used in all experiments.

THP-1, a human acute monocytic leukemia cell line, was obtained from the American Type Culture Collection (Manassas, VA, USA). THP-1 cells were cultured in RPMI-1640 medium (Gibco, NY, USA), supplemented with 10% FBS, 100 IU/mL penicillin, and 100 µg/mL streptomycin.

Cell treatment: HUVECs were allocated into four groups: control (CON), 4-OI, LPS, and 4-OI combined with LPS (4-OI + LPS). Following an overnight incubation, cells were exposed to a serum-free medium for 3 h. Subsequently, the cells in the 4-OI and 4-OI + LPS groups were pretreated with 4-OI (125 µM) exposure for 3 h. Following pretreatment, the initial culture solution was replaced with serum-free medium, and the cells in the LPS and 4-OI + LPS groups were exposed to LPS at a final concentration of 1 µg/mL for varying durations based on the experiment conducted according to the preliminary experimental results. To establish the cell pyroptosis model, ATP (MedChemExpress, USA) was added to the cells and incubated for 2 h prior to protein sample harvest, with a final concentration of 5 mM.

The regents used in this study are listed in Table 1 in the online supplementary materials.

### Cell viability

Cell viability was evaluated with a CCK-8 assay. HUVECs (1 × 10^4^ cells/well) were cultured in 96-well plates. After various treatments, 10 µL of CCK-8 reagent (APExBIO Technology, Houston, TX, USA) was added to each well. Using a microplate reader (BioTek, VT, USA), absorbance at 450 nm was measured to evaluate cell viability.

### Enzyme-linked immunosorbent assay (ELISA)

HUVECs were cultured in 6-well plates at 1 × 10^5^ cells/well. The cells were exposed to 4-OI for 3 h, followed by stimulation with 1 µg/mL LPS for 8 h. Concentrations of TNF-α, IL-6, and IL-1β were examined in the cell-free supernatants using ELISA kits purchased from Mlbio (Shanghai, China).

Levels of TNF-α, IL-6, and IL-1β in mice serum were determined using commercially available ELISA kits (Mlbio, Shanghai, China), following the manufacturer’s instructions.

### DCFH-DA-based cellular ROS detection assay

Cellular reactive oxygen species (ROS) levels were measured with a DCFH-DA probe. HUVECs were cultured in 24-well plates until they reached 90% confluence. Following exposure to 4-OI, LPS, or both, the cells were incubated with 10 µmol/L DCFH-DA for 30 min at 37 ℃ and rinsed three times with PBS to remove residual DCFH-DA. Then, 0.5 mL serum-free medium was added to the cells. Levels of ROS were then measured using fluorescence microscopy (Olympus IX71, Tokyo, Japan) and flow cytometry (Hangzhou Pukang Medical Technology Co., Ltd., China).

### Measurement of MtROS and mitochondrial membrane potential (ΔΨ)

Mitochondrial reactive oxygen species (mtROS) levels were measured using MitoSOX (Yeasen Biotech, Shanghai, China). After the indicated treatments, HUVECs were stained with 5 µM MitoSOX for 10 min. The fluorescent signals emitted by the cells were viewed and documented using fluorescence microscopy (Olympus IX71, Tokyo, Japan).

Mitochondrial membrane potential (ΔΨ) was measured with a JC-1 kit (Beyotime Biotechnology, Shanghai, China). After being incubated in JC-1 staining buffer for 20 min at 37℃, the HUVECs were washed twice. JC-1 monomer (Ex 490 nm/Em 530 nm) and polymer (Ex 525 nm/Em 590 nm) fluorescence intensities were measured with a microplate reader.

### Assessment of mitochondrial morphology

Following treatment, HUVECs were rinsed twice with pre-warmed PBS at 37 °C. Subsequently, the cells were incubated with 125 nM PK Mito Orange (Genevivo, Nanjing, China) in a serum-free medium at 37 °C for 15 min in the dark. Super-resolution structured illumination microscopy (SIM) images were captured using a Multi-SIM X imaging system (NanoInsights-Tech Co., Ltd.), which was equipped with a 63 × 1.46 numerical aperture (NA) oil immersion objective (ZEISS Objective Plan-Apochromat 63 × 1.46 Oil) and a Photometrics Kinetix camera. The imaging was conducted in Low NA GI-SIM mode, utilizing a 50 mW laser power and a 30 ms exposure time. Image reconstruction was performed using the SIM Imaging Analyzer software (NanoInsights-Tech Co., Ltd.). During image acquisition, the cells were maintained in a humidified chamber at 37 °C with 5% CO_2_. Cells exhibiting a network of filamentous mitochondria were categorized as normal, whereas those with fragmented or partially truncated mitochondria were classified as fragmented.

### Measurement of cytosolic MtDNA

The release of mitochondrial DNA (mtDNA) into the cytosol was assessed following previously established protocols (Liu et al. 2022). Briefly, HUVECs were pretreated with 4-OI and subsequently exposed to LPS. Cells were incubated with 1% NP-40 lysis buffer (Beyotime, Shanghai, China) on ice for 15 min. The lysates were collected and centrifuged at 16,215 g for 15 min at 4 °C. The supernatants were moved into the fresh 1.5 mL EP centrifuge tubes and the Genomic DNA was extracted using the TIANamp Genomic DNA Kit (TIANGEN, Beijing, China). Quantification of mtDNA was performed via qRT-PCR using primers specific to the ND-1 gene and the mitochondrial D-loop region. To normalize the data, the levels of a nuclear gene in genomic DNA were measured using primers targeting the 18 S ribosomal RNA gene as a reference standard.

### Monocyte adhesion assay

Prior to performing adhesion assays, HUVECs were treated with 4-OI or LPS as described above. Subsequently, THP-1 cells labeled with 5 µM CMFDA (a live cell tracer; green) (Yeasen Biotechnology, Shanghai, China) were added to confluent HUVECs monolayers. The number of THP-1 cells adhering to the monolayer surface was quantified by fluorescence microscopy (Olympus IX71, Tokyo, Japan).

### Quantitative reverse transcription polymerase chain reaction (qRT-PCR)

RNA was extracted using TRIzol reagent (Vazyme Biotech Co., Ltd, Nanjing, China). cDNA was subsequently generated with the PrimeScript™ RT reagent Kit (Vazyme Biotech Co., Ltd, Nanjing, China). TNF-α, IL-1β, IL-6, MCP-1, ICAM-1, and VCAM-1 levels were measured with a PowerUp™ SYBR™ Green Master Mix Kit (Thermo Fisher Scientific, MA, USA) with a spectrofluorometric thermal cycler (Eppendorf, Hamburg, Germany). The primer sequences (shown in Table 2 in the online supplementary materials) used for PCR were designed with Primer Premier 5.0.

### Endothelial monolayer permeability assay

HUVECs were grown on collagen-coated transwell inserts, which had polycarbonate filters with 0.4 μm pores and a diameter of 6.5 mm (Corning, NY, USA). Once the cells in the upper chambers reached confluence, 4-OI or PBS was added, and left to incubate for 3 h, followed by exposure to LPS for 8 h. A solution of 70 kDa FITC-dextran (0.5 mg/mL) was added to the upper chamber and incubated for 1 h. Media from the lower chambers were then collected, and fluorescence intensity of the FITC-dextran at 488 nm was measured with a spectrophotometer.

### Network Pharmacological analysis of 4-OI against sepsis-induced ALI

The structural data of 4-OI was extracted from PubChem, and the known pharmacological targets of 4-OI were obtained from the SwissTargetPrediction (Daina et al. 2019) and PharmMatch databases (Liu et al. 2010). Similarly, targets associated with sepsis and ALI were retrieved from the GeneCards (Stelzer et al. 2016) and DisGeNET databases. A Venn diagram was generated to overlap the pharmacological targets associated with 4-OI, sepsis, and ALI. An interaction network was built with the shared genes in the STRING database (Szklarczyk et al. 2019), and a network linking the drugs, targets, and diseases was visualized with Cytoscape software. The primary mechanism underlying the therapeutic effects of 4-OI on sepsis-induced ALI was identified as the regulation of genes within the core PPI network. The DAVID database was used to conduct functional annotation with Gene Ontology (GO) and pathway enrichment analyses with Kyoto Encyclopedia of Genes and Genomes (KEGG) of the overlapped genes.

### Molecular Docking of 4-OI to MAPK1 and MAPK8

Molecular docking of 4-OI to its key targets MAPK1 and MAPK8 was performed to determine the potential of 4-OI to modulate these kinases. The structures of MAPK1 and MAPK8 were obtained from the RCSB PDB. The binding energy was calculated with AutoDock Vina (version 1.1.2), with values below − 5.0 kcal/mol suggesting a high binding affinity. The optimal binding mode was visualized with PyMOL software.

Network pharmacological analysis as well as molecular docking related databases and web addresses are provided in Table 3 in the online supplementary materials.

### Western blotting

HUVECs were treated with ice-cold RIPA buffer (Beyotime Biotechnology) supplemented with protease and phosphatase inhibitors and incubated for 30 min. Nuclear protein was extracted using a Nuclear Protein Extraction Kit (Sigma-Aldrich). A BCA protein assay kit was used to measure protein concentrations. Equal amounts of extracted proteins were separated via SDS-PAGE and transferred to an immobilon polyvinylidene fluoride (PVDF) membranes (Merck Millipore, Bayswater, Australia). A solution of 5% skim milk was applied to the membranes and left at room temperature for 1 h, then incubated overnight with specific primary antibodies. Following three washes with TBST, the membranes were incubated with secondary antibodies for 1 h. The protein bands were visualized with a chemiluminescence kit (EpiZyme Scientific, Shanghai, China), and protein expression was quantified with the ImageJ software.

### Immunofluorescence staining and TUNEL assay

For in vitro analysis, HUVECs were fixed with 4% paraformaldehyde for 30 min, permeabilized with 0.1% Triton X-100 for 10 min, and then blocked with 10% fetal bovine serum for 1 h. Subsequently, the cells were incubated with specific primary antibodies overnight at 4 ℃. The following day, a secondary antibody was added and incubated for 1 h, followed by staining with 4,6-diamidino-2 phenylindole (DAPI) for 5 min, and sealed with coverslips.

Cell apoptosis was detected using TUNEL detection kit (Vazyme, Nanjing, China) following the protocol. Imaging was undertaken using a fluorescence microscope (Olympus IX71, Tokyo, Japan).

For lung tissue, immunofluorescence staining was performed on 4 μm sections for histological analysis. The sections underwent antigen retrieval in Tris-EDTA buffer (pH 8.0) in a microwave, followed by incubation with anti-ICAM-1 (1:200) and anti-CD31 (1:200) antibodies. Sections were then treated with secondary antibodies and sealed. Images were captured with a fluorescence microscopy (Olympus IX71, Tokyo, Japan) in a blinded manner.

### Animals

C57BL/6J mice (male, 6–8 weeks old) were obtained from GemPharmatech Co., Ltd. (Chengdu, China) and housed in a facility with a 12-hour light/dark cycle, with free access to food and water. All the animal procedures were approved by the Institutional Animal Care and Use Committee of Southwest Medical University and conformed to the Guide for the Care and Use of Laboratory Animals published by the US National Institutes of Health (NIH Publication No. 85-23, revised 1996). Ethical approval was granted by the Animal Experiment Ethics Committee of Southwest Medical University (No. 20221118-037).

On the experimental day, mice were anesthetized by inhalation of 3% isoflurane (RWD Life Science Co., Ltd, Shenzhen, China) in O_2_ (1 L/min). Mice were then euthanized by exsanguination through cardiac puncture while remaining under anesthesia. Lungs were isolated and dissected for further ex vivo studies, while blood was collected for preparing serum.

### Establishment of mouse sepsis model and treatment

C57BL/6J mice were randomly assigned into four groups (*n* = 6 mice/group). Mice in the 4-OI and 4-OI + LPS groups were administered 4-OI (50 mg/kg) by a single intraperitoneal injection, and mice in the remaining two groups received an equivalent volume of solvent (5% DMSO, 40% PEG300, 5% Tween-80, and 50% saline). After 2 h, mice in the LPS and 4-OI + LPS groups were administered LPS (10 mg/kg) by intraperitoneal injection, while the other two groups received an equivalent volume of vehicle. Studies were conducted after 8 h to LPS or vehicle (Nepal et al. 2019). 

For the survival rate analysis, 40 mice were randomly divided into four groups, with 10 mice per group. A high dose of LPS 20 mg/kg was administered via intraperitoneal injection (Luo et al. 2022). One hour after LPS injection, mice were treated with either 4-OI or solvent. The survival rate was monitored at 12-hour intervals following LPS injection. Survival was observed and calculated from 0 to 5 days using the Kaplan-Meier method.

### Processing of mouse specimens

Mice were anesthetized by inhalation of 3% isoflurane inhaled in O_2_ (1 L/min), blood samples were collected from the inferior vena cava, and the mice were humanely euthanized by exsanguination. After being allowed to settle for 2 h at room temperature, the blood samples were then centrifuged at 1000× g for 20 min. The serum was subsequently separated, divided into aliquots, and stored at -80 ℃ and repeated freeze–thaw cycles avoided. The lungs were excised, and a gross image was captured. They were then fixed in 4% paraformaldehyde overnight, embedded in paraffin, sectioned for further analysis.

### Histomorphology analysis and determination of lung injury score

Paraffin samples were stained with hematoxylin and eosin (H&E) and observed with an upright microscope to assess inflammation and damage in lung tissues. In each section, three randomly selected fields were evaluated for lung injury score in a blinded manner. The scoring system, which has been previously established (Imai et al. 2001; Yamaguchi et al. 2021), assesses five key parameters: alveolar congestion, hemorrhage, neutrophil infiltration, thickness of the alveolar wall, and hyaline membrane formation. The scoring scale is as follows: 0 = minimal (or little) damage, 1 = mild damage, 2 = moderate damage, 3 = severe damage, and 4 = maximal damage. The average lung injury score from the three fields in each section was calculated.

### Wet-to-dry weight ratio of mouse lung

The upper lobe of the right lung was excised, excess connective tissue was removed, and the tissue was gently washed with physiological saline. Excess surface moisture was removed with filter paper, and the wet weight (W) was measured. The tissue was placed in a drying oven at a constant temperature of 60 ℃ for > 72 h until it was completely dehydrated and reached a stable weight, which was recorded as the dry weight (D). The wet-to-dry weight ratio was subsequently calculated.

### Assessment of lung permeability in vivo

8 h after LPS injection, the mice were injected with 1% Evans blue solution (40 mg/kg) through the tail vein. 30 min later, the mice were anesthetized by the inhalation of isoflurane (3% isoflurane with supplemental oxygen) and then euthanized by exsanguination through cardiac puncture under anesthesia. The lung tissues were collected and carefully cleaned with filter paper. Images were captured for documentation purposes. Formamide was introduced at a ratio of 1 mL per 100 mg of lung tissue, and the tube was incubated at 37 ℃ for 24 h to facilitate the reaction. After being centrifuged at 12,000 rpm for 30 min, the supernatant was carefully collected. Optical density (OD) of the supernatant was measured at 620 nm as a measure of permeability.

### Statistical analysis

All the experiments unless otherwise stated were repeated independently three times. Data are expressed as the means ± SDs. Normality was evaluated with the Shapiro-Wilk test. Data from three or more groups were compared with one-way ANOVA followed by Tukey’s post hoc test. Statistical analysis was performed with GraphPad Prism (version 8.0) software, with a *P* value of < 0.05 indicating a significant difference.

## Results

### 4-OI alleviated LPS-induced inflammatory response and oxidative stress in HUVECs

To evaluate the impact of 4-OI on cell viability, HUVECs were treated with 4-OI at various concentrations(0, 31.25, 62.5, 125, 250, 500, and 1000 µM) for different durations(3, 9, and 18 h) (details provided in Supplementary result 1 in the online supplementary materials). The CCK-8 results (online supplementary materials, Figure S1) indicated that exposing HUVECs to 125 µM of 4-OI for 18 h did not adversely affect their viability. Therefore, 4-OI was used at a concentration of 125 µM in all the following experiments.

**Fig. 1:**
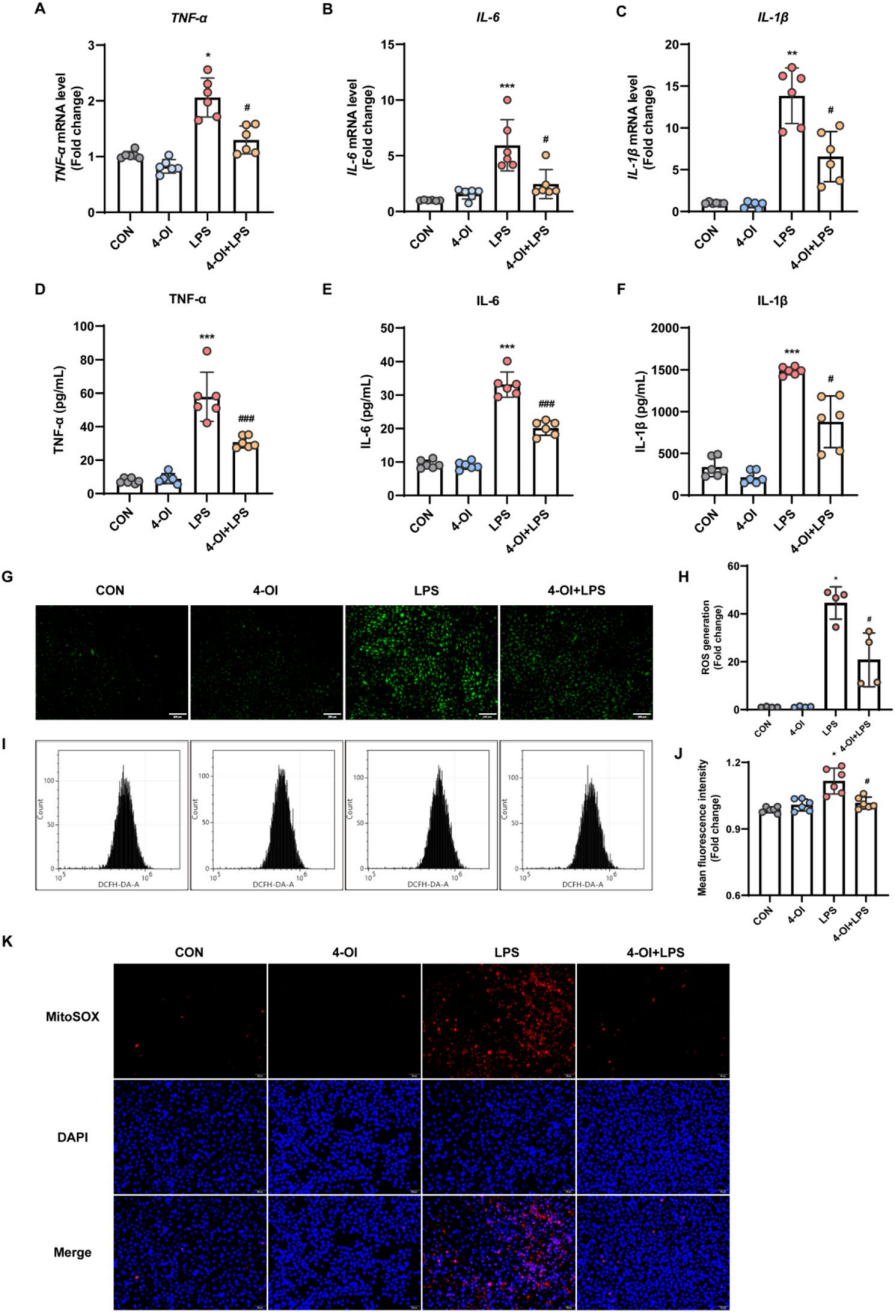
4-OI alleviated LPS-induced inflammatory response and oxidative stress in HUVECs. Cells were pretreated with 4-OI (125 µM) for 3 h and then stimulated with 1 µg/mL LPS. (**A**-**C**) After LPS stimulation, the mRNA expression of the inflammatory cytokines *TNF-α* (**A**), *IL-6* (**B**), and *IL-1β* (**C**) in HUVECs was measured by qRT-PCR (*n* = 6 samples per group). (**D–F**) After 8 h of LPS stimulation, ELISA kits were used to measure the levels of TNF-α (**D**), IL-6 (**E**), and IL-1β (**F**) in the culture supernatants of HUVECs (*n* = 6 samples per group). (**G**-**H**) DCFH-DA, a cell-permeable fluorescent probe, was added to the culture medium at a final concentration of 10 µM. DCFH reacts with ROS in the cells and is metabolized to generate fluorescent DCF. The fluorescence intensity of DCF was observed under a fluorescence microscope (*n* = 4 fields of view per group) (scale bar = 200 μm). (**I**-**J**) Flow cytometry was performed to evaluate the mean fluorescence intensity of DCF in each group, the fold increase in intracellular ROS levels relative to the CON group were visualized on a histogram (*n* = 6 samples per group). (**K**) Representative images of MitoSOX-stained mitochondrial ROS in HUVECs. After the indicated treatments, HUVECs were stained with 5 µM MitoSOX for 10 min, and images were captured with a fluorescence microscope (scale bar = 50 μm). MitoSOX (red), DAPI (blue). The *p* value was generated by one-way ANOVA followed by Turkey’s post hoc test. (^*^P < 0.05, ^**^P <0.01, ^***^P < 0.001 compared with the CON group; ^#^*P* < 0.05, ^###^*P* < 0.001 compared with the LPS group)

The effects of 4-OI on inflammatory cytokines production were measured by analyzing the mRNA levels by qRT-PCR. Figure 1A-C show that 4-OI suppressed *TNF-α*,* IL-6*, and *IL-1β* mRNA levels induced by LPS in HUVECs. ELISA analysis further confirmed the secretion of these proteins was decreased after exposure to 4-OI (mean ± SD: TNF-α, 30.80 ± 3.17 pg/mL vs. 57.85 ± 13.37 pg/mL; IL-6, 20.16 ± 1.98 pg/mL vs. 33.15 ± 3.45 pg/mL; IL-1β, 879.44 ± 282.26 pg/mL vs. 1487.79 ± 43.26 pg/mL), as illustrated in Fig. 1D-F. The results indicate that 4-OI efficiently inhibits the release of inflammatory cytokines in LPS-stimulated HUVECs.

We then determined the effect of 4-OI on ROS production induced by LPS in HUVECs. The fluorogenic probe DCFH-DA was used to assess the generation of ROS in cells (Xiong et al. 2022). The immunofluorescence staining and flow cytometry results revealed a notable increase in cellular ROS level in the LPS group when compared with the control group. 4-OI treatment markedly reduced the fluorescence intensity of DCFH-DA in the LPS group, indicating the ability of 4-OI to suppress the LPS-induced production of cellular ROS (Fig. 1G-J).

The sources of reactive oxygen species (ROS) are diverse, yet mitochondria are considered the primary site of intracellular ROS generation. MitoSOX Red, which fluoresces upon reacting with mitochondrial superoxide, was employed to measure mitochondrial superoxide levels (Johnson-Cadwell et al. 2007; Feng et al. 2022). In HUVECs, LPS treatment significantly increased MitoSOX fluorescence, whereas 4-OI treatment effectively reduced it (Fig. 1K).

### 4-OI preserve mitochondrial function and structure

**Fig. 2:**
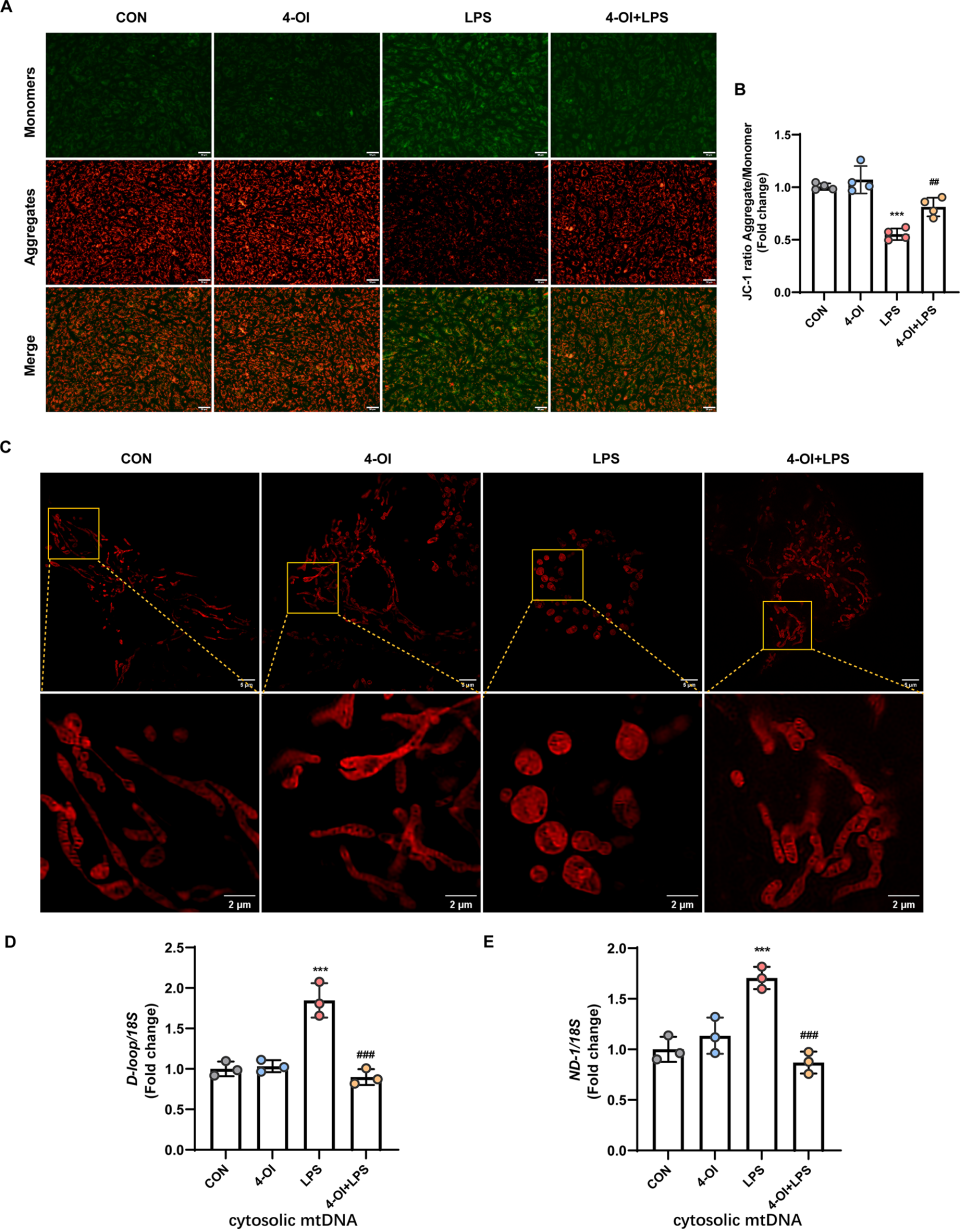
4-OI preserve mitochondrial function and structure. (**A**) HUVECs were treated with 4-OI, stimulated with LPS, and incubated with the JC-1 probe for 20 min, and images were captured with a fluorescence microscope (scale bar = 50 μm). (**B**) The fluorescence intensity of JC-1 monomers (490/530 nm) and aggregates (525/590 nm) was measured with a microplate reader, and the aggregate-to-monomer ratio was calculated (*n* = 4 samples per group). (**C**) Following treatment, HUVECs were washed and incubated with 125 nM PKMito Orange at 37 °C for 15 min in the dark. Super-resolution structured illumination microscopy (SIM) images were captured using a Multi-SIM X imaging system. (Upper: scale bar = 5 μm; Lower: magnified images, scale bar = 2 μm) (**D-E**) qRT-PCR analysis of cytosolic mtDNA (*D-loop*,* ND-1*) normalized to nuclear DNA (*18 S*) in HUVECs (*n* = 3 samples per group). The *p* value was generated by one-way ANOVA followed by Turkey’s post hoc test. (^***^P < 0.001 compared with the CON group; ^*##*^*P* < 0.01, ^*###*^*P* < 0.001 compared with the LPS group)

Inflammatory mediators and oxidative stress can induce changes in the mitochondrial inner membrane and disrupt the electron transport chain, thereby leading to dissipation of the mitochondrial membrane potential (ΔΨ), a critical indicator of mitochondrial health (Liu et al. 2023). The effect of 4-OI on ΔΨ was determined with the JC-1 fluorescent probe. Figure 2A provide representative images for the cells in the CON group exhibited red fluorescent JC-1 aggregates, which are indicative of a hyperpolarized ΔΨ. In contrast, JC-1 aggregates were absent in LPS-treated cells, which showed a prominent shift towards diffuse green fluorescence, suggesting mitochondrial depolarization. These results indicate 4-OI treatment partially reversed the LPS-induced decrease in ΔΨ levels (Fig. 2B).

Changes in ΔΨ are associated with cell apoptosis and mitochondrial membrane damage, which can lead to the release of mitochondrial DNA (mtDNA) into the cytosol. Indeed, LPS exposure resulted in elevated cytosolic mtDNA levels, as indicated by increased levels of *D-loop* and *ND-1* region detected by qRT-PCR, while 4-OI inhibits the release of mtDNA from mitochondria (Fig. 2D-E).

Additionally, mitochondrial morphology was assessed using PK Mito Orange, a mitochondria-specific stain. In the control group, mitochondrial morphology appeared normal, with mitochondria cristae being distinctly visible. In LPS-induced cells, aberrant mitochondrial morphology was characterized by swelling, condensation, and irregular cristae structure. Conversely, these morphological alterations were partially reversed in cells treated with 4-OI, as depicted in Fig. 2C. These results further indicate that 4-OI participates in preserving mitochondrial function and structure.

### 4-OI suppresses HUVECs apoptosis and pyroptosis

**Fig. 3:**
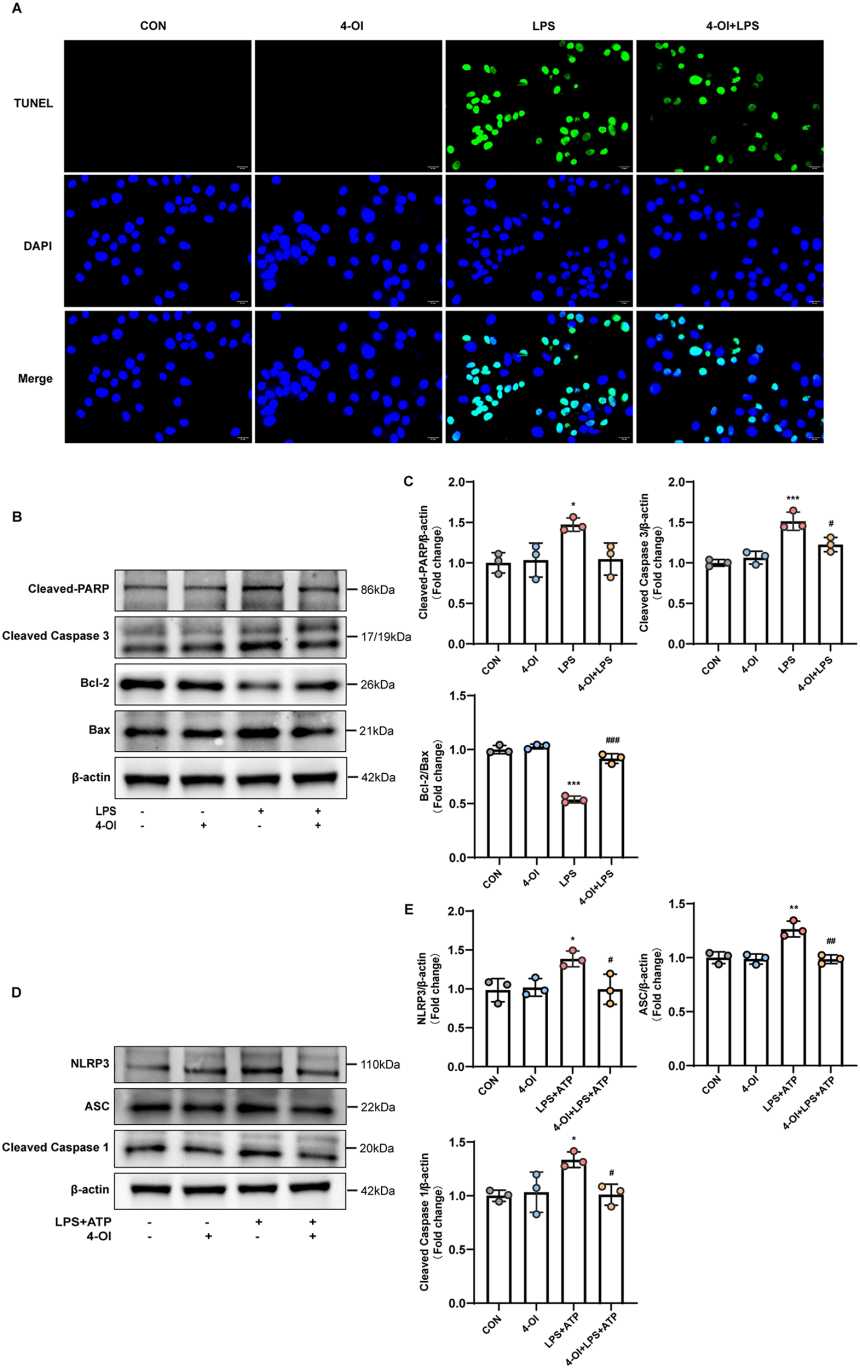
4-OI suppresses HUVECs apoptosis and pyroptosis. (**A**) TUNEL staining was applied to assess the effects of 4-OI on LPS-induced apoptosis of HUVECs. The cells are grown on the coverslip in 6 well plates, treated with 4-OI or LPS or both. The coverslips were fixed with paraformaldehyde followed by precooled ethanol and acetic acid. The coverslips were labeled with TUNEL (green) and counterstained with DAPI nuclear stain (blue). The pictures were taken with a fluorescence microscope (scale bar = 20 μm). (**B**-**C**) Western blotting was used to detect the protein expression of Cleaved-PARP, Cleaved-Caspase 3, Bcl-2, and Bax in whole cell lysates (*n* = 3 samples per group). (**D**-**E**) Western blotting was used to detect the protein expression of NLRP3, ASC, Cleaved-Caspase 1 in whole cell lysates (*n* = 3 samples per group). Representative images of 3 independent experiments are shown and the ration of the density of β-actin normalized to each protein was analyzed (*n* = 3 samples per group). For the above, all data shown are presented as fold changes. Error bar represents the standard deviation and *p* value was generated by one-way ANOVA followed by Turkey’s post hoc test. (^*^P < 0.05, ^**^P < 0.01, ^***^P < 0.001 compared with the CON group; ^#^*P* < 0.05, ^*##*^*P* < 0.01, ^*###*^*P* < 0.001 compared with the LPS+ATP group)

Excessive inflammation and oxidative stress are well-documented triggers of cell apoptosis and pyroptosis. Prior to protein sample collection for western blotting, cells were observed under a light microscopy. Following LPS stimulation, the cells showed a decrease in volume, appearing shrunken, and surrounded by circular small bodies (online supplementary materials, Figure S2A). TUNEL staining was used to evaluate apoptosis in HUVECs. Figure 3A revealed that LPS significantly increased apoptosis, an effect that was effectively mitigated by 4-OI treatment. Notably, 4-OI alone had no significant impact on apoptosis in the control group. Further investigation of apoptosis-related signaling pathways through western blot analysis showed that LPS exposure led to the upregulation of Cleaved-PARP, Caspase-3, and Bax protein levels, while simultaneously downregulating the anti-apoptotic protein Bcl-2. Conversely, 4-OI treatment upregulated Bcl-2 levels and counteracted the LPS-induced changes in Cleaved-PARP, Caspase-3, and Bax, although the effect on Cleaved-PARP was not statistically significance (Fig. 3B-C).

To further investigate the effects of 4-OI on cellular pyroptosis, cells were stimulated with LPS and ATP. This dual stimulation resulted in cell swelling and the formation of multiple bubble-like protrusions on the cell membrane, as observed under a light microscopy (online supplementary materials, Figure S2B). We evaluated the expression levels of key pyroptosis-associated proteins, including NLRP3, ASC, and Cleaved-Caspase 1. The results indicated that exposure to LPS and ATP significantly elevated the protein levels of these markers. However, these effects were effectively mitigated by 4-OI treatment, with 4-OI alone exhibiting minimal impact (Fig. 3D-E).

### 4-OI inhibited LPS-induced monocyte–endothelial cell adhesion

The impact of 4-OI in modulating monocyte adhesion was determined with the THP-1 adhesion assay. Prior to LPS exposure for 4 h, HUVECs were pretreated with either vehicle or 4-OI. The adherence of fluorescence-labelled THP-1 cells (monocytes) to HUVEC monolayers was subsequently determined.

**Fig. 4:**
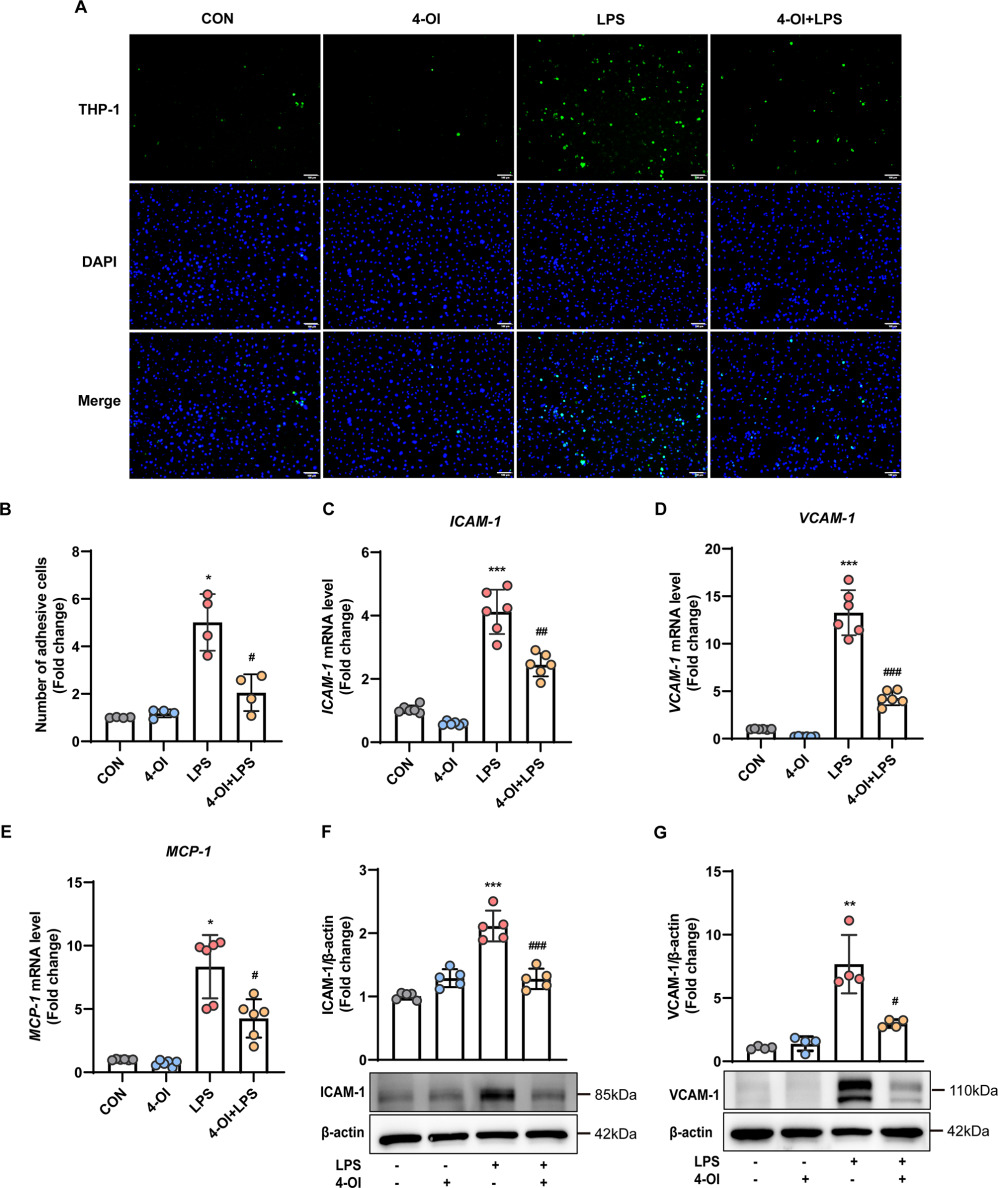
4-OI inhibited LPS-induced monocyte–endothelial cell adhesion. (**A**) Adhesion of THP-1 cells (green) labeled with the live cell tracer CMFDA to HUVECs; the cells were allowed to adhere for 30 min at 37 ℃ and imaged under a microscope (*n* = 4 fields of view per group) (scale bar = 100 μm). (**B**) Quantification of adhesion was normalized to that in the control group (without LPS stimulation). (**C–E**) qRT-RCR was used to measure the mRNA expression of *ICAM-1* (**C**), *VCAM-1* (**D**), and *MCP-1* (**E**) in HUVECs (*n* = 6 samples per group). (**F–G**) Western blotting was used to detect the protein expression of ICAM-1 (**F**) and VCAM-1 (**G**) in whole cell lysates; Representative images of 3 independent experiments are shown and the expression of ICAM-1 and VCAM-1 relative to that of β-actin based on density analysis is shown above the blot (ICAM-1: *n* = 5 samples per group; VCAM-1: *n* = 4 samples per group). For the above, all data shown are presented as fold changes. Error bar represents the standard deviation and *p* value was generated by one-way ANOVA followed by Turkey’s post hoc test. (^*^P < 0.05, ^**^P < 0.01, ^***^P < 0.001 compared with the CON group; ^*#*^*P* < 0.05, ^*##*^*P* < 0.01, ^*###*^*P* < 0.001 compared with the LPS group)

**Fig. 5:**
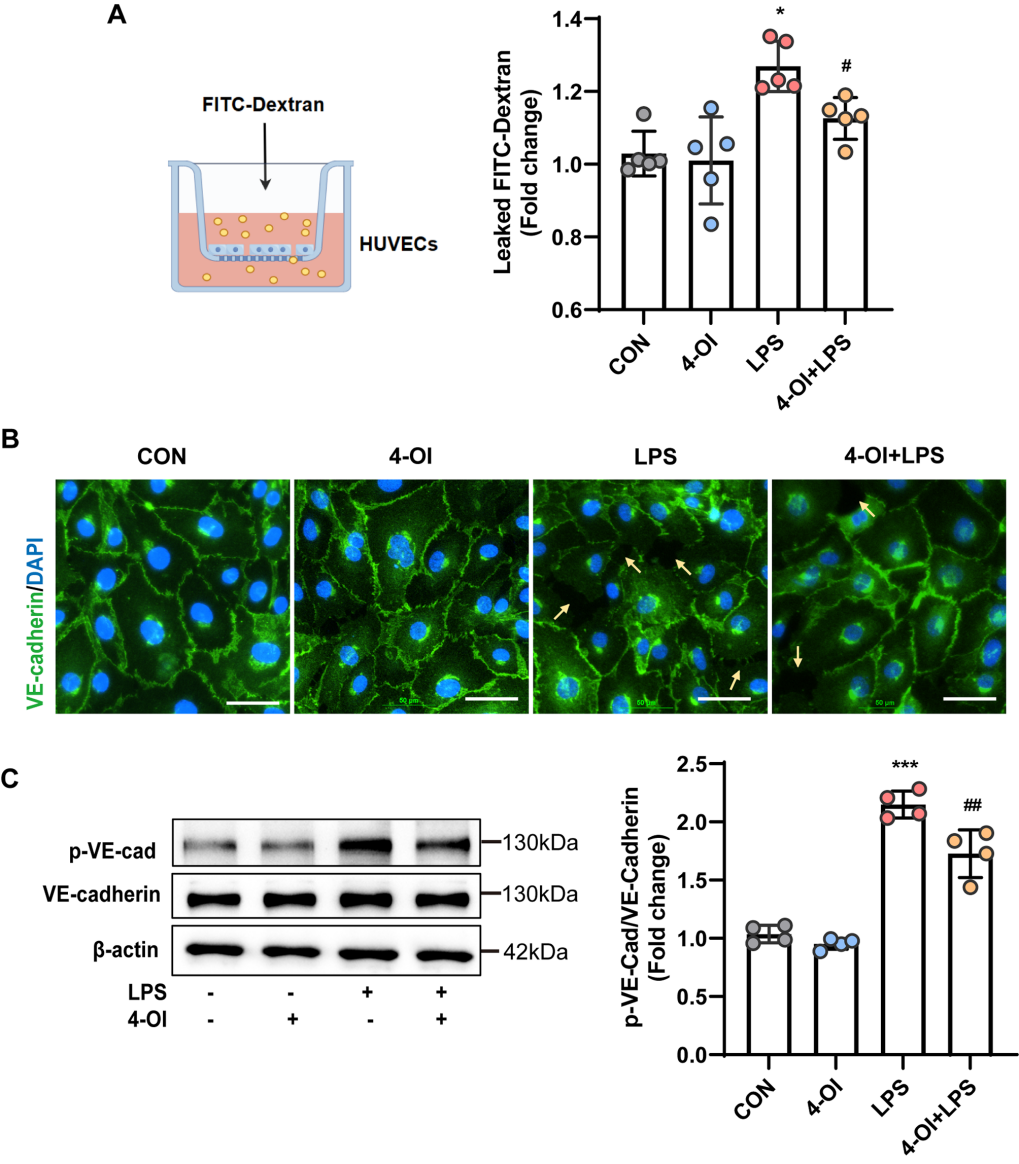
4-OI mitigated the LPS-induced impairment of endothelial barrier integrity in vitro. (**A**) Endothelial permeability was assessed by measuring FITC-Dx-70 flux through HUVEC monolayers within 1 h in a transwell system (*n* = 5 samples per group). (**B**) Representative images of immunostaining for VE-cadherin (green) in HUVECs (*n* = 4 fields of view per group). Nuclei were stained with DAPI (blue) (scale bar = 5 μm). Yellow arrows: impaired inter-cellular adheren junctions between endothelial cells. (**C**) The levels of phospho-VE-cadherin in whole-cell lysates were detected by western blotting, and were normalized to those of VE-cadherin by density analysis (*n* = 4 samples per group). The *p* value was generated by one-way ANOVA followed by Turkey’s post hoc test. (^*^P < 0.05, ^***^P < 0.001 compared with the CON group; ^*#*^*P* < 0.05, ^*##*^*P* < 0.01 compared with the LPS group)

Fig. 4A provided representative images of the cell adhesion assay with summary data and bar graphs shown in Fig. 4B. The group data were shown as the fold change in adhesive cell numbers compared to the control group (first bar) (2.03 ± 0.64-fold in 4-OI + LPS group vs. 4.98 ± 0.95-fold change in LPS group; *P* < 0.05). Due to possible involvement of cytokines and adhesion molecules for monocyte recruitment, we investigated the effect of 4-OI on adhesion molecule expression in HUVECs. The qRT-PCR findings indicated that 4-OI suppressed the LPS-induced upregulation of the cell adhesion molecules *ICAM-1*,* VCAM-1*, and *MCP-1* in HUVECs (Fig. 4C–E). Western blotting analysis confirmed the reduced levels of ICAM-1 and VCAM-1 proteins in HUVECs treated with 4-OI followed by LPS stimulation (Fig. 5F–G). Collectively, 4-OI effectively inhibited the LPS-induced monocyte adhesion to the activated endothelium and reduces the expression levels of adhesion molecules.

### Effects of 4-OI on endothelial barrier integrity

Altered endothelial permeability is considered a hallmark of sepsis, characterized by impaired endothelial barrier integrity resulting from reduced endothelial junction marker expression. This impairment leads to increased permeability to large molecules and the entry of white blood cells into the space beneath the endothelium (Hu et al. 2016). The effect of 4-OI on the permeability of LPS-stimulated HUVECs was assessed with FITC-Dx-70. Figure 5A shows pretreatment with 4-OI effectively mitigated the LPS-induced increase in endothelial permeability.

Additionally, we assessed the expression of endothelial cell junctional adhesion proteins responsible for regulating cell-cell connections, with a particular emphasis on VE-cadherin. In control HUVECs, VE-cadherin exhibited a continuous and uninterrupted expression pattern, as shown in Fig. 5B. LPS exposure disrupted the VE-cadherin surface expression, but pretreatment with 4-OI partially suppressed these changes in cell morphology, resulting in a VE-cadherin expression pattern comparable to that in the CON group.

Furthermore, western blotting analysis revealed a significant decrease in phosphorylated VE-cadherin levels in the 4-OI group compared with the LPS group (Fig. 5C). Taken together, these findings suggest that 4-OI enhances endothelial cell–cell junctions and maintains endothelial barrier integrity.

### 4-OI inhibited LPS-induced TLR4/MAPK signaling pathway activation in HUVECs

**Fig. 6:**
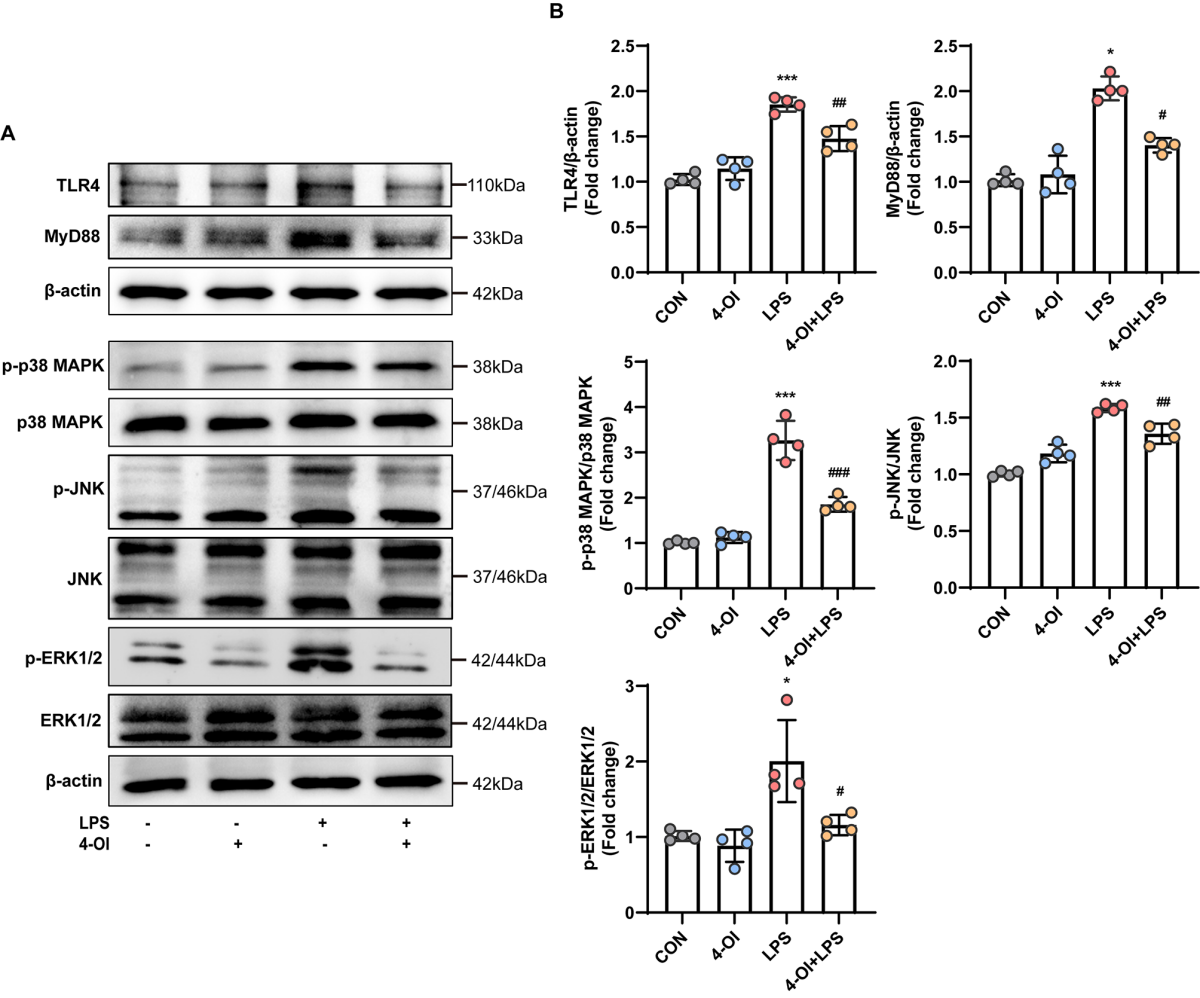
4-OI inhibited LPS-induced TLR4/MAPK signaling pathway activation in HUVECs. (**A**) Western blotting was used to detect the protein expression of TLR4, MyD88, p-p38 MAPK, p-JNK, and p-ERK1/2 in whole cell lysates and representative images are shown. HUVECs in the 4-OI and 4-OI + LPS groups were pretreated with 4-OI exposure for 3 h, followed by LPS stimulation for various durations (TLR4 and MyD88, 24 h; p-ERK1/2, 15 min; p-p38 MAPK and p-JNK, 60 min) (**B**) Relative ratios of the band densities of TLR4/β-actin, MyD88/β-actin, p-p38 MAPK/p38 MAPK, p-ERK1/2/ERK1/2, and p-JNK/JNK (*n* = 4 samples per group). The *p* value was generated by one-way ANOVA followed by Turkey’s post hoc test. (^*^P < 0.05, ^***^P < 0.001 compared with the CON group; ^*#*^*P* < 0.05, ^*##*^*P* < 0.01, ^*###*^*P* < 0.001 compared with the LPS group)

Numerous studies (Zheng et al. 2024; Lan et al. 2023) have demonstrated the critical role of the TLR4/MAPK pathway in LPS-induced production of inflammatory mediators, which are key elements in the progression of sepsis. According to the findings from network pharmacology and molecular docking results, the MAPK signaling pathway was identified as a possible candidate for 4-OI ameliorating sepsis (online supplementary materials, Figure S3A-E). Consequently, we determined the effect of 4-OI on the TLR4/MAPK signaling cascade. As expected, exposure to LPS led to the increased expression of TLR4, MyD88 and the activation of important MAPK pathway components (p-p38 MAPK, p-JNK, and p-ERK1/2). Pretreatment with 4-OI reduced the protein levels of TLR4, MyD88and suppressed the activation of p38 MAPK, JNK, and ERK1/2 in HUVECs stimulated with LPS (Fig. 6A-B).

### Effects of 4-OI on the NF-κB signaling pathway

**Fig. 7:**
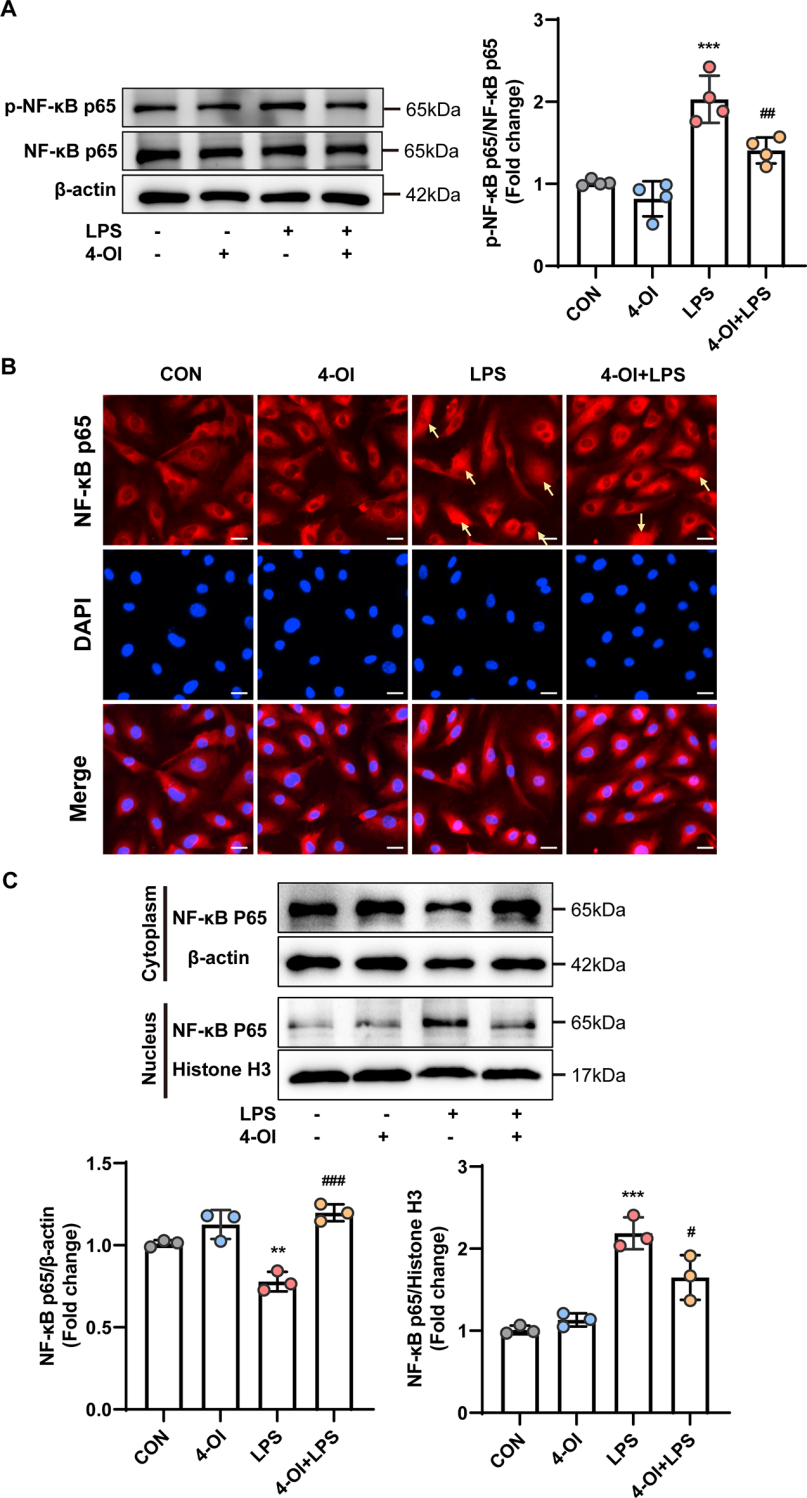
4-OI inhibited LPS-induced NF-κB signaling pathway activation in HUVECs. (**A**) The protein expression of p-NF-κB p65 in whole cell lysates was measured by western blotting and normalized to that of NF-κB p65 by density analysis (*n* = 4 samples per group). (**B**) Immunofluorescence analysis of the nuclear translocation of NF-κB p65 in HUVECs after 1 h of LPS stimulation. The arrow indicates the translocation of NF-κB p65 from the cytoplasm to the nucleus (*n* = 4 samples per group) (scale bar = 20 μm). (**C**) Western blotting was performed to detect the protein expression of NF-κB p65 in the cytoplasm and nucleus, with β-actin and histone H3 serving as the internal references in the cytoplasm and nucleus, respectively (*n* = 3 samples per group). The *p* value was generated by one-way ANOVA followed by Turkey’s post hoc test. (^**^P < 0.01, ^***^P < 0.001 compared with the CON group; ^*#*^*P* < 0.05, ^*##*^*P* < 0.01, ^*###*^*P* < 0.001 compared with the LPS group)

NF-κB is an essential component of the MAPK signaling cascade and substantially contributes to cellular inflammatory responses. Consequently, we examined the effects of 4-OI on NF-κB signaling, with a specific focus on the phosphorylation and nuclear translocation of NF-κB p65. The results indicated that the LPS-induced increase in p-NF-κB p65 was partly reversed by 4-OI (Fig. 7A). Immunofluorescence staining revealed that NF-κB p65 was located in the cytoplasm in the CON group, whereas it was in the nucleus in the LPS group. Notably, 4-OI inhibited the translocation of NF-κB p65 into the nucleus (Fig. 7B). Furthermore, the inhibitory effects of 4-OI on the LPS-induced nuclear translocation of NF-κB p65 were confirmed by western blotting (Fig. 7C). Overall, these findings suggest that 4-OI successfully regulates inflammation by suppressing the TLR4/MAPK/NF-κB pathway, thereby exerting a protective effect on the endothelial cell barrier.

### 4-OI alleviated lung injury and improved overall survival in sepsis-induced ALI mice

**Fig. 8:**
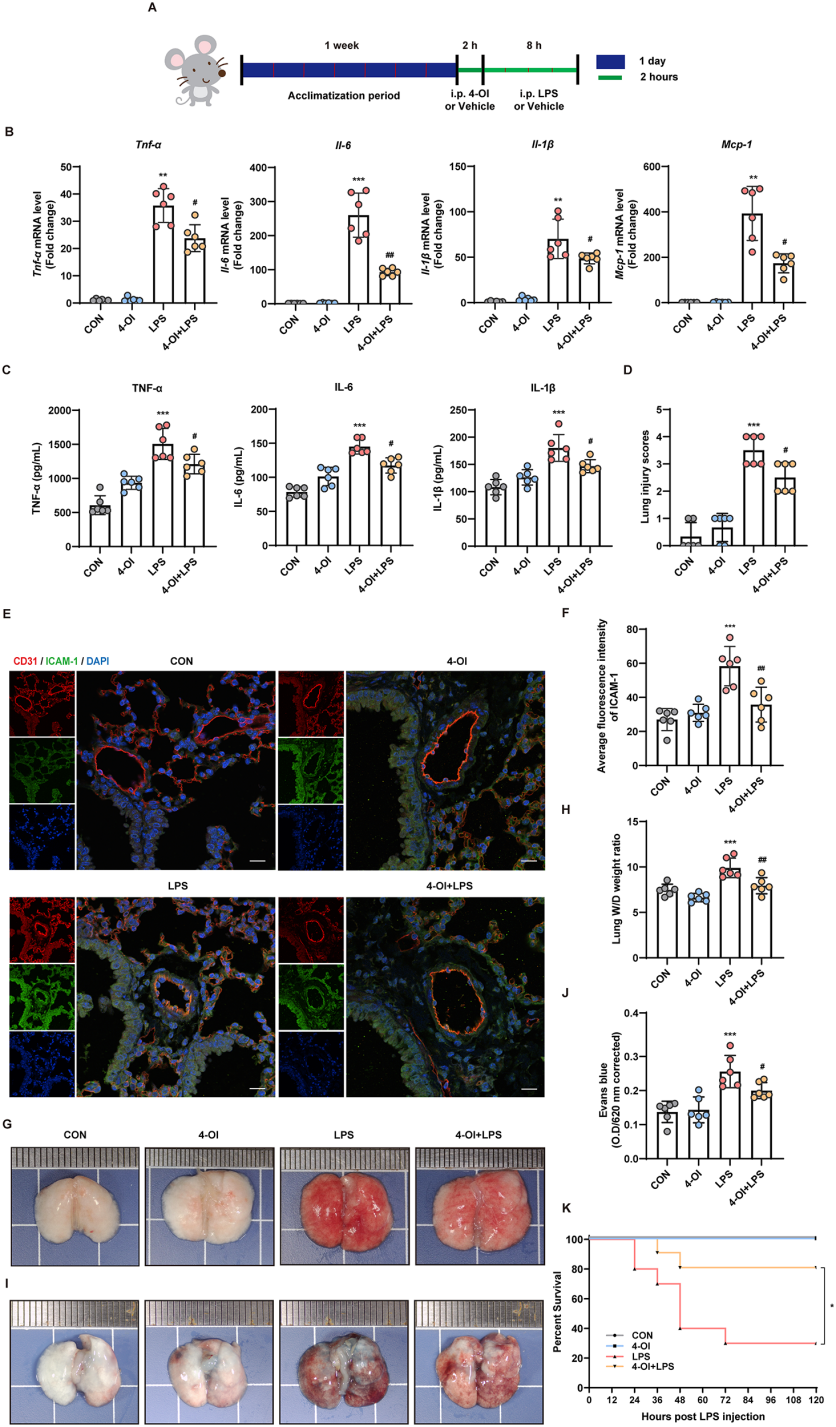
4-OI alleviated lung injury and improved survival in sepsis-induced ALI mice. **(A)** Schematic diagram of the in vivo experimental design. **(B)** qRT-PCR was performed to measure the relative mRNA expression of *Tnf-a*, *Il-6*, *Il-1β*, and *Mcp-1* in mouse lung tissues (*n* = 6 mice/group). **(C)** ELISA was performed to detect the protein levels of TNF-α, IL-1β, and IL-6 in mouse serum (*n* = 6 mice/group). **(D)** Semiquantitative analysis of lung injury in each group (*n* = 6 mice/group). **(E-F)** Immunofluorescence co-staining for CD31 (red) and ICAM-1 (green) in pulmonary vascular endothelial cells (*n* = 6 mice/group). Cell nuclei were stained with DAPI (blue) (scale bar = 200 μm). **(G)** Images of mouse lung tissues from each group (*n* = 6 mice/group). **(H)** The dissected lung tissues were weighed and dried at 60 ℃ for 72 h, and the wet weight -dry weight (W/D) ratio was calculated (*n* = 6 mice/group). **(I)** Images of mouse lung tissues after the injection of Evans blue. **(J)** Statistical analysis of the results of Evans blue staining using a microplate reader after formamide extraction (*n* = 6 mice/group). (**K**) Induction of ALI by a high dose of LPS (20 mg/kg, i.p.). The mortality of the mice was monitored every 12 h, and the percent survival rate was expressed as a Kaplan-Meier survival curve (*n* = 10 mice per group). The *p* value was generated by one-way ANOVA followed by Turkey’s post hoc test. (^*^P < 0.05, ^**^P < 0.01, ^***^P < 0.001 compared with the CON group; ^*#*^*P* < 0.05, ^*##*^*P* < 0.01 compared with LPS group in A-I; * *P* < 0.05 compared with the LPS group in K)

A mouse model of LPS-induced sepsis was established to investigate the effects of 4-OI on sepsis-induced vascular injury and ALI in vivo. After 1 week of acclimatization, the mice in the treatment group were administered with 4-OI 2 h prior to the LPS injection, whereas those in the control group received the same volume of solvent. Subsequently, the mice received an intraperitoneal injection of LPS (10 mg/kg) to induce sepsis-induced ALI (Fig. 8A).

The results in Fig. 8B-C indicated that 4-OI effectively suppressed LPS-induced inflammatory reactions, resulting in decreased mRNA levels of *Tnf-α*, *Il-6*, *Il-1β*, and *Mcp-1* in the lung tissues of the mice and decreased protein levels of TNF-a, IL-1β, and IL-6 in the serum of the mice (mean ± SD: TNF-α, 1210.56 ± 131.27 pg/mL vs. 1506.40 ± 207.64 pg/mL; IL-6, 117.36 ± 10.20 pg/mL vs. 145.04 ± 9.87 pg/mL; IL-1β, 146.84 ± 10.88 pg/mL vs. 180.20 ± 22.35 pg/mL). H&E staining revealed that, compared with the CON group, the LPS group exhibited significant inflammation in lung tissues, including infiltration with inflammatory cells, damage to alveolar walls, and lung congestion. However, pretreatment with 4-OI notably suppressed these changes (online supplementary materials, Figure S4).

Figure 8D illustrates that the lung injury score was notably lower in the 4-OI group than that in the LPS group. Immunofluorescence staining for CD31 (an endothelial cell marker) and ICAM-1 demonstrated that 4-OI attenuated the LPS-induced upregulation of ICAM-1 in mouse pulmonary vascular endothelial cells (Fig. 8E-F). The results suggest that administration of 4-OI effectively reduces inflammatory responses within the lung tissues of mice suffering from sepsis-induced ALI.

Furthermore, the impact of 4-OI on pulmonary oedema and vascular endothelial barrier integrity was investigated. As shown in Fig. 8G, the mice in the LPS group exhibited more pronounced hemorrhage in lung tissues compared to mice in the 4-OI group. Additionally, the lung water content, as assessed by measuring the wet-to-dry weight ratio, was significantly increased in the LPS group than that in the 4-OI + LPS group (Fig. 8H).

The impact of 4-OI on vascular barrier function was examined by evaluating changes in pulmonary capillary permeability by Evans blue staining. The findings revealed a significant increase in the distribution of Evans blue in the lung tissues of mice from the LPS group. However, treatment with 4-OI inhibited the vascular injury caused by LPS (Fig. 8I-J). These findings suggest that 4-OI protected against lung damage by maintaining the functional integrity of the vascular endothelial barrier. 

To investigate the effects of 4-OI on the long-term survival rate of septic mice, a survival analysis was conducted using Kaplan-Meier analysis. Mice were administered a high dose of LPS (20 mg/kg, n = 10 mice/group). 1 h post-injection, the mice were treated with either 4-OI or solvent. The survival curves were then compared using the log-rank Mantel-Cox test. In the absence of LPS, both the control and 4-OI groups demonstrated comparable 100% survival rate, suggesting that 4-OI does not exhibit significant toxicity. However, upon LPS challenge, the 4-OI treatment group exhibited significantly enhanced resistance to LPS-induced sepsis, resulting in prolonged survival compared to the LPS-only group (Fig. 8K).

## Discussion

Sepsis frequently leads to ALI, characterized by the disruption of the lung endothelial and pulmonary epithelial barriers (Zhou et al. 2019). Despite substantial research efforts, the current treatment for ALI predominantly involves supportive measures such as mechanical ventilation, which still results in a mortality rate of 40% even with advanced intensive care unit (ICU) interventions (Lee et al. 2020; Cheng et al. 2017). This situation underscores the urgent need for therapeutic strategies aimed at stabilizing the pulmonary endothelial barrier. The present study illustrates that 4-OI confers protective effects against sepsis-induced vascular injury and organ damage by attenuating endothelial inflammation, oxidative stress, and barrier dysfunction. These findings underscore the therapeutic potential of 4-OI in the management of sepsis and provide novel insights into the molecular mechanisms underlying its protective effects.

We employed LPS to stimulate HUVECs as an in vitro model of cellular injury to test our hypothesis. Our findings demonstrated that 4-OI markedly inhibited the production of LPS-induced pro-inflammatory cytokines (TNF-α, IL-6, IL-1β) in HUVECs. This observation is consistent with previous studies indicating the role of itaconate derivatives in modulating immune responses in inflammatory conditions (Bambouskova et al. 2018; Mills et al. 2018). Notably, the suppression of the TLR4/ MAPK/ NF-κB signaling pathway was identified as a pivotal mechanism, corroborating its established involvement in sepsis-associated endothelial activation (Akira and Takeda 2004; Liu et al. 2017). By inhibiting MAPK phosphorylation and preventing NF-κB nuclear translocation, 4-OI likely disrupted the amplification of inflammatory pathways, thereby mitigating cytokine storms, a characteristic feature of sepsis progression (Hotchkiss and Karl 2003). 

A significant finding was the ability of 4-OI to maintain mitochondrial homeostasis, as indicated by decreased mitochondrial reactive oxygen species (mtROS) accumulation, stabilization of mitochondrial membrane potential, and prevention of mitochondrial DNA (mtDNA) release. Mitochondrial dysfunction is increasingly recognized as a critical factor in endothelial injury during sepsis, precipitating both apoptosis and pyroptosis (Zorov et al. 2014; Poll et al. 2017). The observed decrease in TUNEL-positive cells, along with and the inhibition of apoptosis and pyroptosis-related markers further indicates that 4-OI confers protection to endothelial cells via dual anti-apoptotic and anti-pyroptotic mechanisms (Galluzzi et al. 2018; Shi et al. 2017). This stabilization of mitochondria may further elucidate the reduction in oxidative stress and inflammatory responses, considering the interaction between mitochondrial damage and the activation of the NLRP3 inflammasome (Zhou et al. 2011). 

The ability of 4-OI to maintain endothelial barrier integrity is of equal importance. By inhibiting the expression of ICAM-1/VCAM-1 and suppressing the phosphorylation and internalization of VE-cadherin, 4-OI effectively reduces leukocyte adhesion and vascular leakage, which are critical factors in sepsis-induced organ injury (Dejana et al. 2008; Ley et al. 2007). The preservation of junctional complexes, along with reduced FITC-Dx-70 permeability, highlights its role in mitigating endothelial hyperpermeability—a pathological feature closely associated with mortality in septic patients (Ince et al. 2016). These in vitro results were validated in vivo, where pretreatment with 4-OI alleviated pulmonary edema, vascular leakage, and histopathological damage in mice challenged with LPS (Rittirsch et al. 2009). Importantly, the observed survival benefit, even when administered post-sepsis, indicates a clinically relevant therapeutic window that warrants further investigation (Singer et al. 2016). 

While our findings contribute to the advancement of current understanding, several critical questions remain unresolved. Firstly, the specific identity of endothelial membrane receptors responsible for mediating endogenous itaconate signaling requires further elucidation. Additionally, comparative studies are necessary to ascertain whether macrophage-derived endogenous itaconate produces effects on endothelial cells equivalent to those observed with exogenous supplementation. Although we have demonstrated the involvement of the TLR4/MAPK/NF-κB pathway, the intricate interactions involving the HIF-1α and STING pathways in the context of vascular injury (Evans et al. 2022; He et al. 2023) necessitate concurrent investigation. Furthermore, the therapeutic potential of 4-octyl itaconate (4-OI) necessitates additional pharmacokinetic profiling, including dose optimization and the assessment of off-target effects, despite its demonstrated benefits in enhancing post-sepsis survival.

In conclusion, this study identifies 4-OI as a multifaceted agent in combating sepsis-induced vascular injury, effectively targeting both the inflammatory and structural aspects of endothelial dysfunction. By elucidating its mitochondrial protective and barrier-stabilizing properties, our findings enhance the understanding of itaconate biology and provide a rationale for the development of 4-OI-based therapies for sepsis and associated vascular pathologies.

## Electronic supplementary material

Below is the link to the electronic supplementary material.

Supplementary Material 1

## Data Availability

No datasets were generated or analysed during the current study.
