# Supplementary material for: 4-Octyl itaconate alleviates endothelial cell inflammation and barrier dysfunction in LPS-induced sepsis via modulating TLR4/MAPK/NF-κB signaling: 4-Octyl itaconate alleviates endothelial dysfunction
Source: Mol Med. 2025 Jun 16;31:240. doi: 10.1186/s10020-025-01160-2 (PMC12168283; doi:10.1186/s10020-025-01160-2)
Supplement: Supplementary file 1 — Supplementary Material 1 [file 10020_2025_1160_MOESM1_ESM.docx]

**Supplementary Material**

1. **Octyl Itaconate alleviates endothelial inflammation and barrier dysfunction in LPS-induecd septis via modulating TLR4/MAPK/**

**NF-κB signaling**

Rong Li ^1†^, Yu Ma ^1†^, Haoran Wu ^2†^, Xiao Zhang ^3^, Nianhui Ding ^4^, Zhichao Li ^1^, Xin Hu ^1^, Jiajia Rao ^3^, Yiting Zhou ^3^, Liqun Wang ^1^, Ying Wan ^3^, Yan Yang ^5^, Jianbo Wu ^1*^, Xiaoqin Zhang ^3^, Chunxiang Zhang ^6*^

**Supplemental Methods**

**Table 1. Reagents**

| **Name** | **Company** |
| --- | --- |
| 4-Octyl Itaconate | MedChemExpress, New Jersey, USA |
| LPS  ATP | Sigma, St. Louis, MO, USA  MedChemExpress, New Jersey, USA |
| NFκB p65, phospho-NFκB p65 | Cell Signaling Technology, Beverly, MA, USA |
| p38MAPK, phospho-p38MAPK | Cell Signaling Technology, Beverly, MA, USA |
| ERK1/2, phospho-ERK1/2 | Cell Signaling Technology, Beverly, MA, USA |
| VE-cadherin | Cell Signaling Technology, Beverly, MA, USA |
| Phospho-VE-cadherin | Affinity Biosciences, Cincinnati, OH, USA |
| ICAM-1 | Santa Cruz, Dallas, TX, USA |
| VCAM-1 | Abcam, Cambridge, MA, USA |
| MyD88 | Abcam, Cambridge, MA, USA |
| JNK, phospho-JNK | Proteintech, Shanghai, China |
| TLR4  Cleaved-PARP  Cleaved-Caspase 3  Bcl-2  Bax  NLRP3  ASC  Cleaved-Caspase 1 | Proteintech, Shanghai, China  Cell Signaling Technology, Beverly, MA, USA  Abmart, Shanghai, China  Abmart, Shanghai, China  Abmart, Shanghai, China  Proteintech, Shanghai, China  Cell Signaling Technology, Beverly, MA, USA  Affinity Biosciences, Changzhou, China |
| β-actin | Proteintech, Shanghai, China |
| HRP-labeled goat anti-rabbit IgG(H+L) | Beyotime Biotechnology, Shanghai, China |
| HRP-labeled goat anti-mouse IgG(H+L) | Beyotime Biotechnology, Shanghai, China |
| RIPA lysis buffer  NP-40 lysis buffer | Beyotime Biotechnology, Shanghai, China  Beyotime Biotechnology, Shanghai, China |
| BCA protein assay kit | Beyotime Biotechnology, Shanghai, China |
| FITC-Dextran  Nuclear Protein Extraction Kit  PK Mito Orange  TIANamp Genomic DNA Kit  MitoSOX Red Mitochondrial Superoxide Indicator  TUNEL BrightGreen Apoptosis Detection Kit | Yeasen Biotechnology, Shanghai, China  Solarbio Science & Technology, Beijing, China  Genvivo Biotechnology, Nanjing, China  Tiangen Biotechnology, Beijing, China  Yeasen Biotechnology, Shanghai, China  Vazyme Biotechnology, Nanjing, China |

| **Gene** | **Forward primer** | **Reverse primer** |
| --- | --- | --- |
| **Human** | | |
| IL-1β | ATGATGGCTTATTACAGTGGCAA | GTCGGAGATTCGTAGCTGGA |
| IL-6 | ACTCACCTCTTCAGAACGAATTG | CCATCTTTGGAAGGTTCAGGTTG |
| TNF-α | CCTCTCTCTAATCAGCCCTCTG | GAGGACCTGGGAGTAGATGAG |
| MCP-1 | CAGCCAGATGCAATCAATGCC | TGGAATCCTGAACCCACTTCT |
| ICAM-1 | ATGCCCAGACATCTGTGTCC | GGGGTCTCTATGCCCAACAA |
| VCAM-1 | GGGAAGATGGTCGTGATCCTT | TCTGGGGTGGTCTCGATTTTA |
| 18s  ND1  D-loop | CGGCTACCACATCCAAGGAA  CACCCAAGAACAGGGTTTGT  CTATCACCCTATTAACCACTCA | GCTGGAATTACCGCGGCT  TGGCCATGGGTATGTTGTTAA  TTCGCCTGTAATATTGAACGTA |
| **Mouse** | | |
| Il-1β | GCAACTGTTCCTGAACTCAACT | ATCTTTTGGGGTCCGTCAACT |
| Il-6 | TAGTCCTTCCTACCCCAATTTCC | TTGGTCCTTAGCCACTCCTTC |
| Tnf-α | CCCTCACACTCAGATCATCTTCT | GCTACGACGTGGGCTACAG |
| Mcp-1 | TTAAAAACCTGGATCGGAACCAA | GCATTAGCTTCAGATTTACGGGT |
| 18s | GTTCTTAGTTGGTGGAGCGATTT | AGGGCATCACAGACCTGTTATTG |

**Table 2. The primer sequences used for PCR**

**Table 3. Network pharmacological analysis and molecular docking related databases and web addresses**

| **Database** | **Web address** |
| --- | --- |
| PubChem | https://pubchem.ncbi.nlm.nih.gov/ |
| SwissTargetPrediction | http://www.swisstargetprediction.ch/ |
| PharmMatch | https://www.lilab-ecust.cn/pharmmapper/ |
| GeneCards | https://www.genecards.org/ |
| DisGeNET | https://www.disgenet.org/ |
| STRING | https://cn.string-db.org/ |
| RCSB PDB | https://www.rcsb.org/ |

**Supplemental Results**

**Supplementary result 1. Effects of 4-OI on cell viability**

The structural formula of 4-OI is shown in Supplementary Figure 2A. To evaluate the impact of 4-OI on cell viability, HUVECs were treated with 4-OI at varying concentrations (0, 31.25, 62.5, 125, 250, 500, and 1000 μM) for 3 hours, 9 hours, and 18 hours. CCK-8 result showed no obvious differences among groups after 3 hours of treatment. However, treatment with 4-OI at concentrations of 250 and 500 μM exerted cytotoxic effects after 9 and 18 hours (Supplementary Figure 2B–D). These results suggested that exposing HUVECs to 125 μM of 4-OI for 18 hours did not adversely affect their viability. Consequently, 4-OI was used at the maximum concentration of 125 μM in subsequent experiments.

**
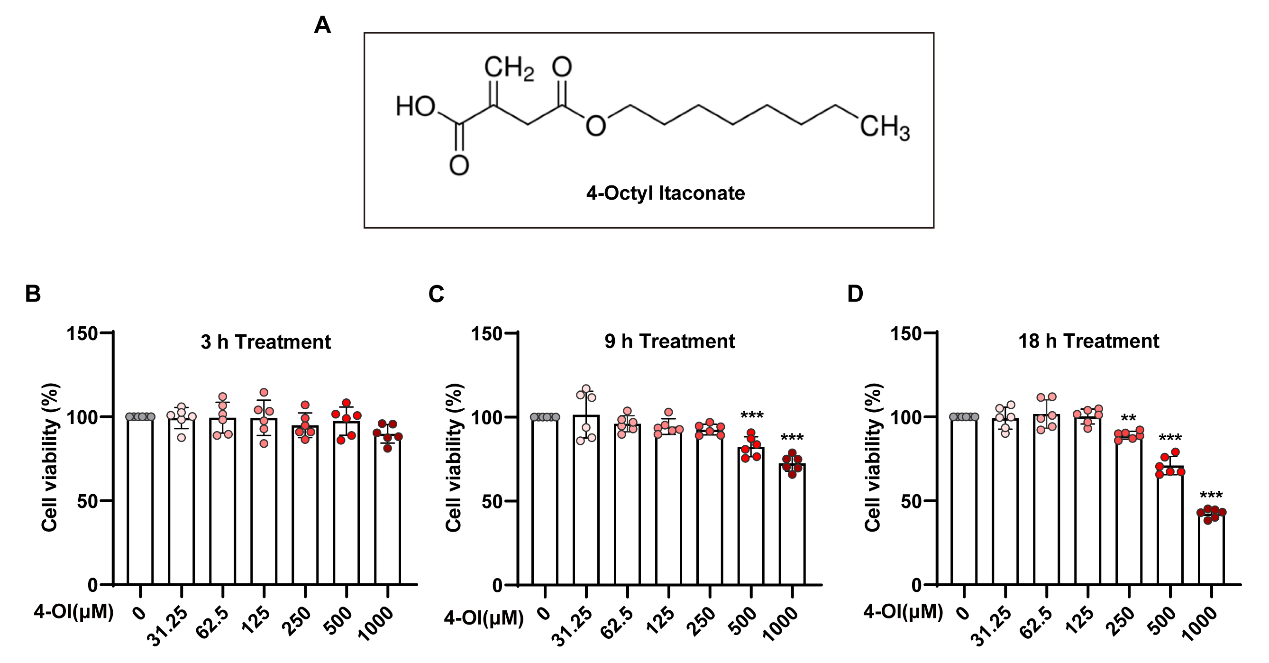
**

**Supplementary Figure 1. Effects of 4-OI on cell viability. (A)** Structural formula of 4-OI. **(B–D)** HUVECs were treated with the indicated concentrations of 4-OI for 3 hours (B), 9 hours (C), and 18 hours (D), and cell viability was evaluated using CCK-8 assay (n = 6). The *p* value was generated by one-way ANOVA followed by Turkey’s post hoc test. (^**^, *P* < 0.01; ^***^, *P* < 0.001 compared with the 0 μM group).

**Supplementary result 2. Effects of 4-OI on 4-OI suppresses HUVECs apoptosis and pyroptosis**

Prior to protein sample collection for western blotting, cells were observed under a light microscopy. Following LPS stimulation, the cells showed a decrease in volume, appearing shrunken, and surrounded by circular small bodies (online supplementary materials, Figure S2A). Upon stimulation with both LPS and ATP, the cells displayed swelling and the formation of multiple bubble-like protrusions on the cell membrane (online supplementary materials, Figure S2B).


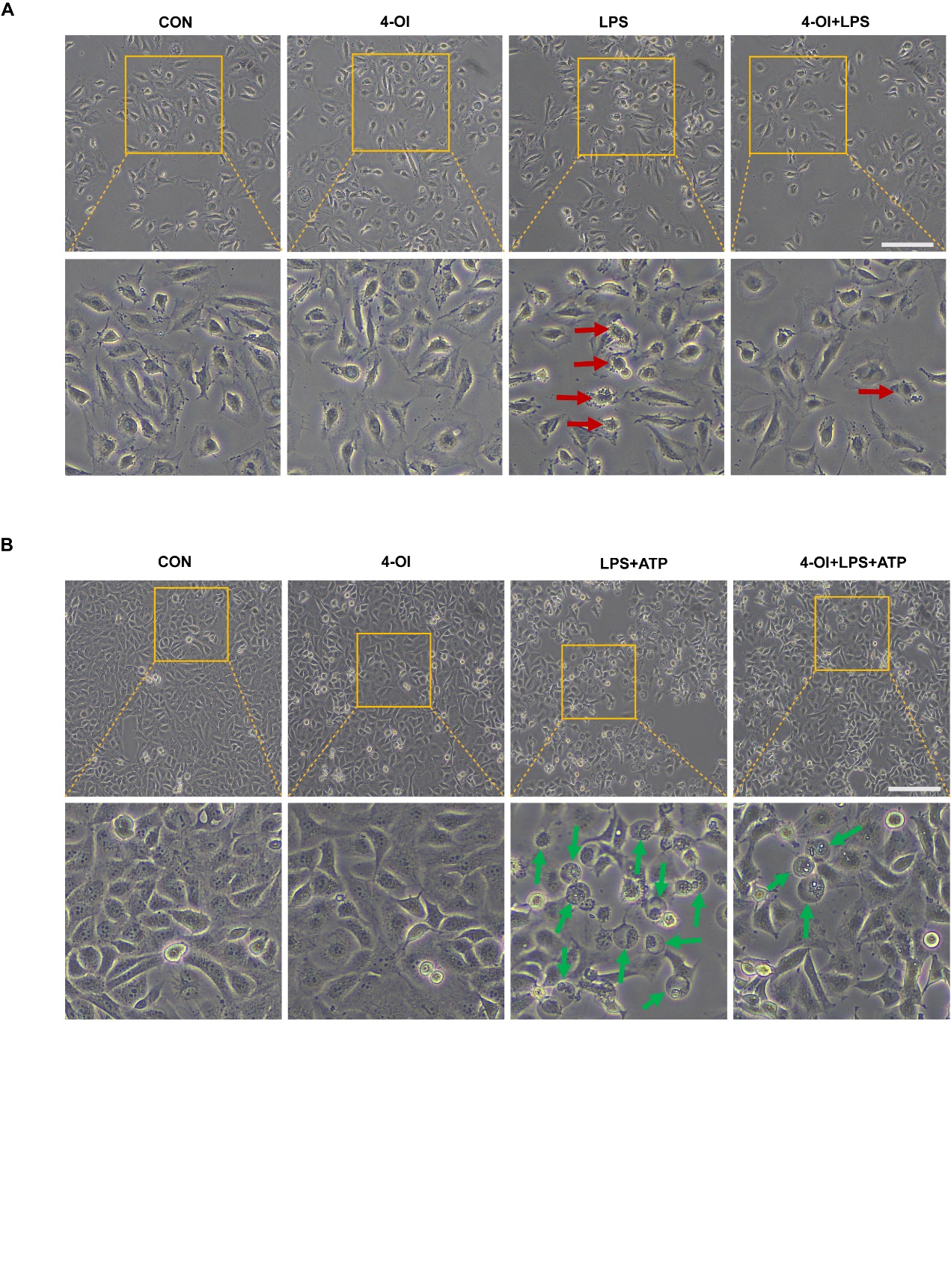


**Supplementary Figure 2. 4-OI suppresses HUVECs apoptosis and pyroptosis. (A)** Representative images of cell morphology changes following LPS challenge, captured by light microscopy (scale bar = 50 μm). Red arrow: impaired cells; **(B)** Representative images of cell morphology changes following LPS and ATP challenge, captured by light microscopy (scale bar = 50 μm)**.** Green arrow: impaired cells

**Supplementary result 3. Network pharmacological analysis and molecular docking**

**S3.1 Target Prediction and Screening of 4-OI against sepsis-induced ALI**

Network pharmacological analysis was performed to elucidate the mechanism of action of 4-OI in the treatment of sepsis. A total of 362 pharmacological targets of 4-OI, 2377 sepsis-related genes and 1139 ALI-related genes were obtained from online databases. As shown in the Venn diagram in Supplementary Figure 3A, 86 overlapping genes were identified as potential targets of 4-OI against sepsis-induced ALI.

**S3.2 PPI network of targets of 4-OI against sepsis-induced ALI**

To investigate the interactions between the targets of 4-OI against sepsis, the 164 overlapping genes were imported into the STRING database to construct a PPI network. The network consisting of 86 nodes with an average degree of 27.1.

In the PPI network, each node represented a target gene, with larger node sizes indicating higher degree values. The interaction strength was expressed as the number of node connections. Topological analysis based on the degree values of nodes was used to identify key targets in the PPI network. The top 23 targets were screened based on the two-fold median value (Supplementary Figure 3B). The top 23 targets included AKT1, ALB, CASP3, EGFR, MMP9, PTGS2, BCL2, HSP90AA1, SRC, PPARG, IGF1, MMP2, IL2, JAK2, CASP1, CCL5, MAPK1, KDR, RHOA, ANXA5, ACE, GSK3B, MAPK8. These genes may serve as key targets of 4-OI against acute lung injury associated with sepsis.

**S3.3 GO and KEGG enrichment analyses**

GO and KEGG enrichment analyses were performed to determine the biological functions of the top 20 overlapping target genes. GO analysis indicated that the genes were primarily associated with biological processes such as response to lipopolysaccharide, regulation of MAP kinase activity, protein phosphorylation, cellular response to reactive oxygen species etc. (Supplementary Figure 3C). KEGG pathway enrichment analysis was performed to predict the signaling pathways influenced by 4-OI. The findings revealed that the top 20 overlapping target genes were predominantly enriched in PI3K-Akt signaling pathway, IL-17 signaling pathway, MAPK signaling pathway etc. (Supplementary Figure 3D). These findings are crucial for elucidating the pathogenesis of sepsis-induced ALI and identifying potential therapeutic targets.

**S3.4 Molecular docking of 4-OI to the key protein in the MAPK signaling pathway**

We selected the key target MAPK1 and MAPK8 for molecular docking owing to its close relationship with inflammation and its involvement in the MAPK signaling pathway as indicated by the results of GO and KEGG analyses. The structure of MAPK1 and MAPK8 was obtained from RCSB PDB. The results of molecular docking showed that the binding energy of the 4-OI–MAPK1 and 4-OI–MAPK8 complex were both less than -5.0 kcal/mol, indicating a strong binding affinity (Supplementary Figure 3E). Given that the binding of a ligand to its receptor is crucial for its biological function, regulation of the inflammatory response via the MAPK signaling pathways may represent a key mechanism of action of 4-OI in the treatment of sepsis-induced ALI.

**
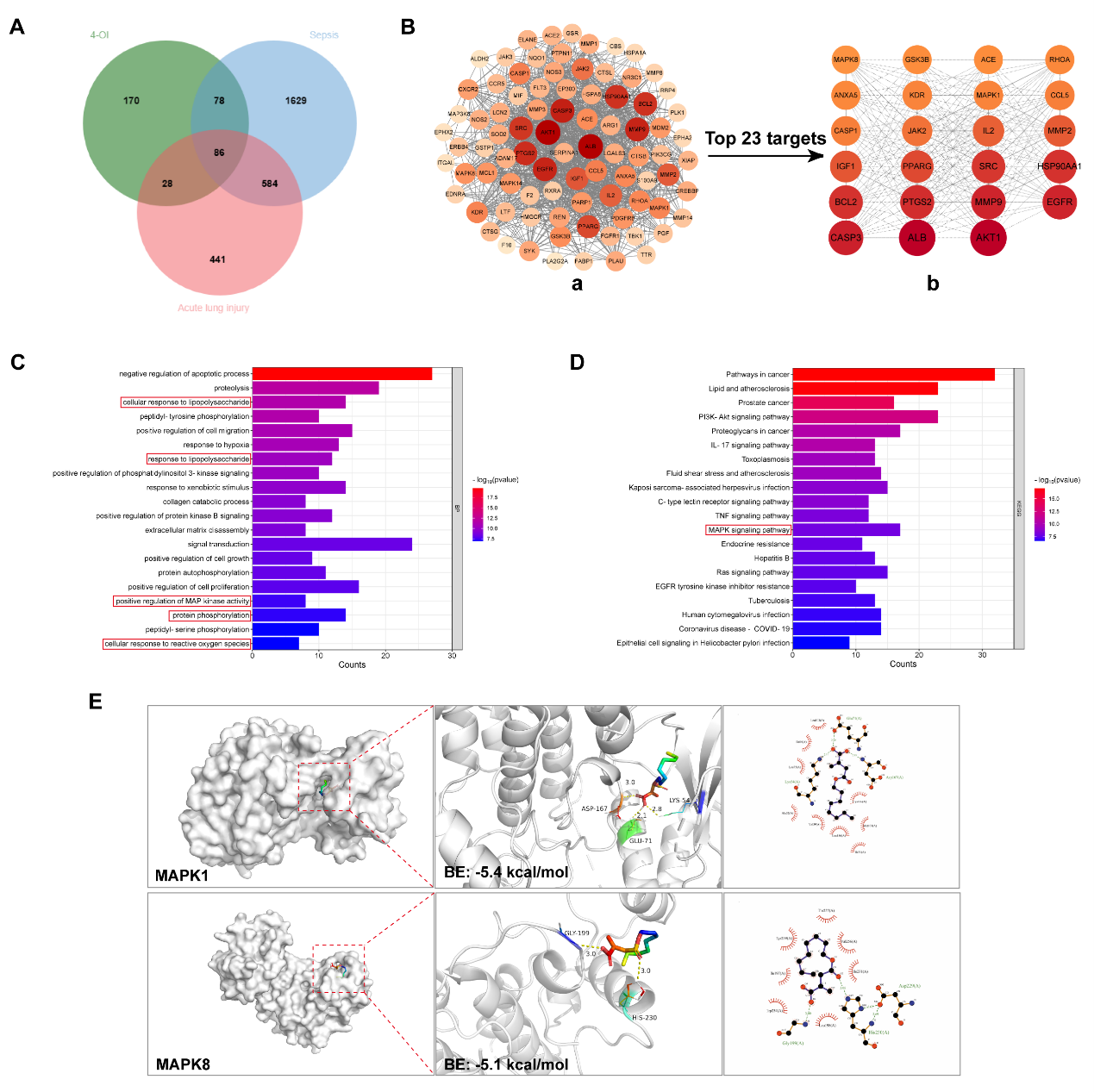
**

**Supplementary figure 3. Network pharmacological analysis of the targets and mechanisms of action of 4-OI. (A)** Venn diagram demonstrating the intersection between targets of 4-OI, sepsis-related and ALI-related genes. **(B)** Protein–protein interaction (PPI) network of potential targets of 4-OI against. a: Interactive PPI network of common targets between 4-OI and sepsis-induced ALI. b: PPI network of targets of 4-OI against sepsis-induced ALI identified from a. A positive relationship was observed between the node size and degree. **(C)** Top 20 GO terms associated with the potential targets of 4-OI against sepsis-induced ALI. **(D)** Top 20 KEGG pathways associated with the potential targets of 4-OI against sepsis. Color coding indicates different thresholds for adjusted p-values, and the length of the band represents the number of genes associated with each term. **(E)** Molecular docking of the key targets MAPK1 and MAPK8 to 4-OI, with the active compounds represented by ball-and-stick models and the secondary structure of the proteins represented by ribbons.

**Supplementary Figure 4**

**
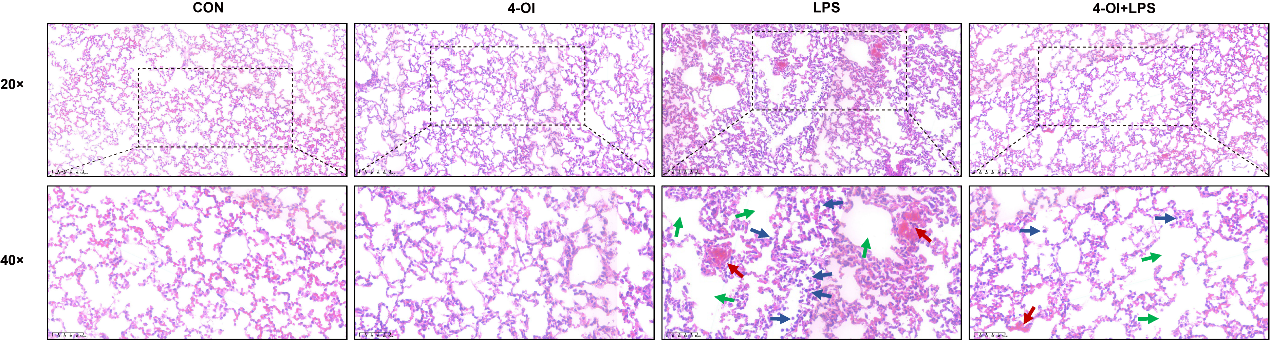
**

**Supplementary Figure 4.** Representative images of H&E staining of lung tissues examined using a light microscope (n = 6 mice/group) (20×:scale bar = 100 μm; 40×: scale bar = 50 μm). Blue arrow: inflammatory cell infiltration; Green arrow: alveolar wall damage; Red arrow: lung congestion
